# TRAIL receptors promote constitutive and inducible IL-8 secretion in non-small cell lung carcinoma

**DOI:** 10.1038/s41419-022-05495-0

**Published:** 2022-12-15

**Authors:** Francesca Favaro, Fedra Luciano-Mateo, Joaquim Moreno-Caceres, Miguel Hernández-Madrigal, Demi Both, Chiara Montironi, Franziska Püschel, Ernest Nadal, Eric Eldering, Cristina Muñoz-Pinedo

**Affiliations:** 1 Preclinical and Experimental Research in Thoracic Tumors (PReTT), Molecular Mechanisms and Experimental Therapy in Oncology Program (Oncobell), Institut d’Investigació Biomèdica de Bellvitge (IDIBELL), L’Hospitalet de Llobregat, 08908 Barcelona, Spain; 2 Amsterdam UMC location University of Amsterdam, Department of Experimental Immunology, Meibergdreef 9, Amsterdam, The Netherlands; 3 Thoracic Oncology Unit, Department of Medical Oncology, Institut Català d’Oncologia (ICO), L’Hospitalet de Llobregat, 08908 Barcelona, Spain; 4 Amsterdam Institute for Infection and Immunity, Cancer Immunology, Amsterdam, The Netherlands; 5 Cancer Center Amsterdam, Cancer Biology, Amsterdam, The Netherlands

**Keywords:** Non-small-cell lung cancer, Mechanisms of disease

## Abstract

Interleukin-8 (IL-8/CXCL8) is a pro-angiogenic and pro-inflammatory chemokine that plays a role in cancer development. Non-small cell lung carcinoma (NSCLC) produces high amounts of IL-8, which is associated with poor prognosis and resistance to chemo-radio and immunotherapy. However, the signaling pathways that lead to IL-8 production in NSCLC are unresolved. Here, we show that expression and release of IL-8 are regulated autonomously by TRAIL death receptors in several squamous and adenocarcinoma NSCLC cell lines. NSCLC constitutively secrete IL-8, which could be further enhanced by glucose withdrawal or by treatment with TRAIL or TNFα. In A549 cells, constitutive and inducible IL-8 production was dependent on NF-κB and MEK/ERK MAP Kinases. DR4 and DR5, known regulators of these signaling pathways, participated in constitutive and glucose deprivation-induced IL-8 secretion. These receptors were mainly located intracellularly. While DR4 signaled through the NF-κB pathway, DR4 and DR5 both regulated the ERK-MAPK and Akt pathways. FADD, caspase-8, RIPK1, and TRADD also regulated IL-8. Analysis of mRNA expression data from patients indicated that IL-8 transcripts correlated with TRAIL, DR4, and DR5 expression levels. Furthermore, TRAIL receptor expression levels also correlated with markers of angiogenesis and neutrophil infiltration in lung squamous carcinoma and adenocarcinoma. Collectively, these data suggest that TRAIL receptor signaling contributes to a pro-tumorigenic inflammatory signature associated with NSCLC.

## Introduction

Interleukin-8 (IL-8) is a small chemokine of the CXC subtype. It is associated with cancer development, particularly in the lung, to the point that mRNA and blood protein levels of IL-8 correlate with tumor burden and poor prognosis in lung cancer patients [1–3]. The roles of IL-8 in lung cancer progression are manifold. IL-8 stimulates tumor cell proliferation [4] and promotes angiogenesis via the recruitment of endothelial cells to the tumor microenvironment [5, 6]. Additionally, its pro-inflammatory actions are linked to immune evasion. The best characterized inflammatory roles of IL-8 in lung cancer are neutrophil and myeloid-derived suppressor cell (MDSC) attraction [7, 8], as well as Neutrophil Extracellular Trap (NET) formation [7, 9], which might be involved in metastatic spread [10].

IL-8 is induced in response to pro-inflammatory stimuli such as viral infection, IL-1, or activation of Toll-like receptors [11], by growth factors like EGF [12] or by environmental stresses such as nutrient deprivation or hypoxia [13, 14]. Other IL-8 inducers are the death ligands, which include Tumor Necrosis Factor alpha (TNFα), Fas/CD95 ligand, and (TNF)-related apoptosis-inducing ligand (TRAIL). Upon stimulation, IL-8 secretion is predominantly controlled at the transcriptional level by NF-κB and JNK or MEK MAP kinases, and by the stress-induced factor ATF4 [13, 15]. The expression of IL-8 under non-stimulated conditions is, however, not as well studied as upon treatment with inflammatory stimuli. IL-8 is elevated in virtually every lung cancer patient, and Non-Small Cell Lung Carcinoma (NSCLC) cell lines secrete it in the absence of any stimuli. Its expression is higher in *KRAS* or *EGFR* mutant cell lines, where levels of the mRNA of IL-8 (gene name *CXCL8*) are ten to a hundred times higher than in non-transformed cells [16].

Here, we investigated the pathways that regulate IL-8 production in human lung cancer cells and that could potentially be targeted in cancer patients. We identified several IL-8-regulating kinases known to be activated downstream of death receptors. Surprisingly, we found an involvement of the TRAIL receptors DR4 and DR5 in IL-8 production in the absence of TRAIL. In the past couple of decades, the death ligand TRAIL received great attention as a possible inducer of cancer-specific cell death, but more recently, it has been shown that death ligands have dual functions as promoters of cell death or inducers of inflammation [17–20]. This inflammation could be pro-tumorogenic, and indeed the murine TRAIL receptor can drive tumorigenesis in a model of NSCLC [18, 21]. We therefore studied the mechanisms by which the chemokine IL-8 is produced and secreted in a tonic manner in NSCLC cell lines and which of these pathways are driven by TRAIL receptors.

## Materials and methods

### Cell lines and treatments

A549, H2126, H460, SW900 (Non-small cell lung cancer, NSCLC), HeLa (cervical cancer), and LLC-1 (murine Lewis Lung carcinoma-1) cells were cultured in pyruvate-free high-glucose DMEM (25 mM) (Gibco, Life Technologies) supplemented with 10% FBS (Life Technologies) and 2 mM L-glutamine (Life Technologies) and incubated at 37 °C in a 5% CO_2_ atmosphere. For glucose deprivation treatment, cells were washed twice with FBS-, pyruvate- and glucose-free DMEM and then treated with this supplemented with 10% dialyzed FBS (1% dFBS when indicated) and 2 mM of L-glutamine, while the control cells were supplemented with 25 mM fresh glucose (Sigma). For drug experiments, compounds were freshly added in media prepared as previously described. As positive control, cells were stimulated with TNFα (Peprotech) or SuperKiller TRAIL (Enzo LifeSciences) at a concentration of 10 and 50 ng/ml, respectively. For blocking the transcription machinery, Actinomycin D (MedChemExpress) was used at 80 nM. Different inhibitory drugs were employed in this study: Necrostatin-1s (RIPK1 inhibitor; Enzo LifeScience); GSK872 (RIPK3 inhibitor; Calbiochem); Takinhib (Tak-1 inhibitor, Sigma); PD98059 (MEK inhibitor; Calbiochem); Trametinib (MEK inhibitor; Selleckchem) Bay11-7082 (NF-κB inhibitor, Calbiochem); SB203580 (p-p38 and Akt inhibitor, Enzo LifeScience); AZD8055 (mTOR inhibitor; Selleckchem); MK2206 (Akt1/2/3 inhibitor; Selleckchem). For hypoxia treatment, cells were located for 24 h in the hypoxic chamber at 0.1% O_2_. All cell lines were used in the passage range between 1 and 25. All cell lines were tested mycoplasma-negative and authenticated by Eurofins Genomic Europe Applied Genomics GmbH using PCR-single-locus-technology.

### DNA constructs

pSI-Check2-hRluc-NFκB-firefly, a reporter plasmid for NFκB, was purchased from AddGene (plasmid number #106979), while an empty vector was used as control, received from Buschbeck’s lab. Cells were transfected as described below using the reverse transfection.

### Transfections and CRISPR knockouts

For siRNA, cells were seeded together with transfection mixture of 100 nM, 1 mg/ml of Lipofectamine 2000 Transfection Reagent (Invitrogen), and DMEM without supplements following the manufacturer’s instructions. For silencing of DR4, DR5, cells were washed twice with FBS-, pyruvate-, and glucose-free DMEM after 24 h incubation with transfection mixture and treated with DMEM in presence (25 mM) or absence (0 mM) of glucose. Same for silencing of TRADD, RIPK1, Casp8, FADD, and TRAIL but after 48 h incubation with the transfection mixture.

For plasmid transfection, cells were seeded together with the transfection mixture of 1 mg of indicated plasmid DNA, 1 mg/ml of Lipofectamine 2000 Transfection Reagent, and DMEM without supplements. After 16 h incubation with transfection mixture, media was replaced with DMEM in presence (25 mM) or absence (0 mM) of glucose, or with glucose and supplemented with TNFα (10 ng/ml).

DR4 and DR5 knockout (KO) were generated in A549 cells using single guide RNAs cloned in the lentiCRISPRv2 (for DR5) and in the Lenti-guide-hygro-dTomato (for DR4). Using HEK 293 T cells, lentiviruses were produced and used to infect A549 cells. Selection in 5 µg/ml puromycin (for DR5) or 500 µg/ml hygromycin (for DR4). DR5 KO cells underwent single-cell cloning. KO was confirmed by Western blot.

### Luciferase reporter assay

Transcriptional activation of NFκB in A549 and HeLa cells was measured using the Dual-Luciferase Reporter assay by Promega. Luciferase activity was detected by Victor^TM^ X Multilabel reader by Perkin Elmer. For each experiment, for each condition Firefly and Renilla luminescence detection was measured in triplicate on black 96-well plate. Relative levels of luciferase activity were calculated normalizing the levels of luciferase activity to the Renilla control plasmid and then to the levels of the control samples.

### Gene expression analysis by qPCR

Cells were trypsinized and pelleted for 5 min at 1200 rcf at RT. RNA extraction was performed using the PureLink RNA Mini Kit (Invitrogen, ThermoFisher) following the manufacturer’s instructions. One microgram of RNA for each sample was retrotranscribed to cDNA using the High Capacity cDNA Reverse Transcription Kit (Applied Biosystems, Invitrogen) following manufacturer’s instructions. For each experiment, qPCR reactions were set up in duplicate and run using the LightCycler 480 SYBR green (Roche). Reactions were prepared with 10 ng of cDNA, 1 mM of primer mixture (forward and reverse), and PowerUp SYBER Green Master mix (Applied Biosystems, Invitrogen). CT values were determined and normalized to L32 as housekeeping gene.

### Western blots

Cells were collected and lysed in RIPA buffer (ThermoFisher) supplemented with protease and phosphatase inhibitors (Roche). After sonication, proteins were quantified by the BCA assay Kit (Pierce, Cultek). Thirty micrograms of proteins per sample were loaded in 12% acrylamide gel. Proteins were transferred to nitrocellulose membranes (Bio-Rad) which were blocked for 1 h in 5% non-fat dry milk in TBS-T (0.1 M Tris-HCl; 1.5 M NaCl; 0.1% Tween-20 in H_2_O, pH 7.5). Depending on the antibody, membranes were incubated with primary antibody for 2 hours at RT or O/N at 4 °C in 2.5% milk in TBS-T. Primary antibodies were diluted 1:1000 except for β-actin which was 1:2000. Membranes were developed using freshly prepared ECL reagent (Promega) with the Amersham 600 imager (Life Science) or with the Odyssey XF imager, Li-Cor (Biosciences). Proteins bands were then quantified using ImageJ and values were normalized to β-actin. Fold change was calculated normalizing towards each control.

### ELISA

Supernatants were collected and centrifuged at 3000 rcf to remove dead cells and debris. According to manufacturer’s instructions, supernatants were diluted in blocking buffer to be in the optimal optical range and analyzed by ELISA using the DuoSet ELISA Ancillary Reagent Kit (R&D Systems, Biotechne). Optical densities were measured with PowerWave XS microplate spectrophotometer (BioTek Instruments) at 450 and 540 nm. Final cytokine concentrations (pg/ml) are displayed as fold change of the control in the figures. When stated pg/mg, final cytokine concentrations are multiplied per volume in culture, then normalized to the protein amount of each sample (measured by BCA assay as described above for WB) and finally displayed as fold change of the control in the figures.

### Quantitative DNA protein interaction (qDPI)-ELISA

qDPI-ELISA was performed as described elsewhere [22]. Briefly, A549 cells were plated and treated with the indicated media. Cells were collected and nuclei were isolated.

In parallel, forward and reverse ss-oligonucleotide sequences 3’-biotin-tagged for *CXCL8* promoter were annealed and coated on a Streptavidin-coated clear 96-well plate (ThermoFisher Technologies) for 2 h at RT. Nuclear proteins were then loaded onto each well, in a final concentration of 25 mg/well, with addition of extra poly dI/dC (1 mg/ml), and incubated O/N at 4 °C. Wells were then washed and blocked with streptavidin wash buffer for 30 min at RT. Primary and HRP-secondary antibodies were incubated at RT. Wells were finally washed and incubated with TMB substrate in dark. Reaction was stopped, and absorbance was measured at 450 nm using PowerWave XS microplate spectrophotometer. Data are displayed as fold change of the OD versus the CTRL sample.

### TRAIL receptor localization

After the indicated treatment, cells were trypsinized and 1 × 10^6^ cell/ml per condition used for each staining. Surface staining was performed with diluted DR4-APC and DR5-FITC in PBS 1X + 0.5% BSA (PBA) at 4 °C for 20 min. Subsequently, cells were fixed and permeabilized at 4 °C for 30 min using a fixation/permeabilization concentrate, following the manufacturer’s instructions (eBioscience™, Invitrogen). A second staining (intracellular) for DR4 and DR5 was performed to half of the wells, and the rest were incubated with PBA 1×, for 20 min at 4 °C. DR4 and DR5 amounts were measured by FACS. A549 DR4 KO and DR5 KO were used as negative controls. Data are presented as average of geometric mean fluorescence intensity (gMFI) of each replicate. Intracellular staining was calculated subtracting the surface gMFI value (first staining only) from the total gMFI (both staining) value.

### Cell death measurement

Cells were plated and treated with the indicated media for 24 h. Cells were then collected by trypsinization together with the supernatants and the PBS used for washing, in order to include floating/dead cells. These were pelleted and resuspended in 100 µg/ml Propidium Iodide (PI) in PBS 1× and measured by FACS. Data are displayed as percentage of viable cells (PI negative).

### Statistical analysis

Statistical analyses were performed using a (Ratio) paired t-test between conditions, or one-way ANOVA test using Dunnett’s multiple comparison corrections; using GraphPad Prism, version 9. *p*-value: *p < 0.05, **p < 0.01, ***p < 0.001.

Bioinformatics analysis was performed using cBioportal [23, 24]. The dataset used for LUSC and LUAD correlation expression analysis is TCGA, Firehose Legacy dataset.

### Supplemental materials

Full list of antibodies is provided in Supplementary Table 1 and oligonucleotide sequences are described in Supplementary Table 2.

Uncropped western blots are provided as Supplementary Materials: Uncropped WB.

## Results

### Lung cancer cell lines with different genetic backgrounds secrete IL-8 in nutrient-deprived and nutrient-rich conditions

**Fig. 1:**
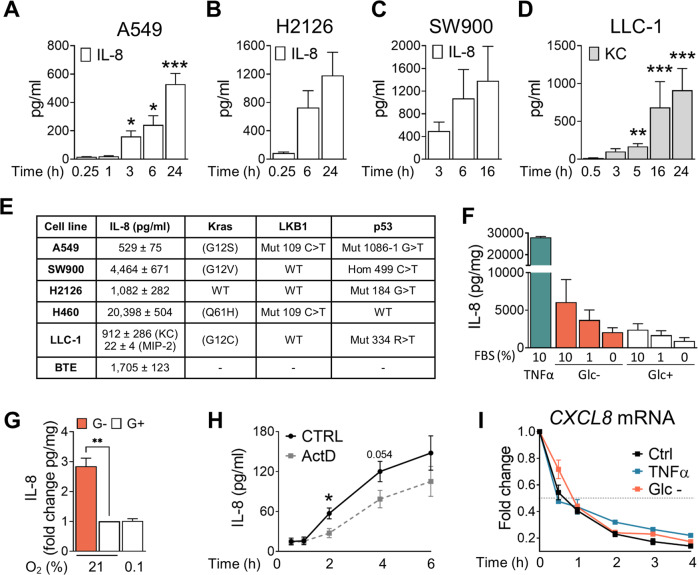
Lung cancer cell lines with different genetic backgrounds secrete IL-8 in nutrient-poor and nutrient-rich conditions. **A** A549 cells were plated and incubated in media containing glucose 25 mM supplemented with 10% dialyzed FBS for the times shown. IL-8 protein concentration was measured by ELISA. Results are expressed as Mean ± SEM of at least 4 experiments. **B**, **C** H2126 and SW900 cells were plated and incubated for the indicated time points. IL-8 was measured by ELISA. Results are expressed as Mean ± SEM (*N* = 3). **D** Lewis Lung Carcinoma cells were seeded and the supernatant was collected at indicated time points to analyze the IL-8 orthologue KC by ELISA. Results are expressed as Mean ± SEM (*N* = 3). **E** Table representing significant mutations of the lung cell lines and the amount of IL-8 they secrete 24 h after medium replacement. **F** A549 cells were plated and incubated in media with different concentrations of dialyzed FBS (dFBS), with TNFα (10 ng/ml) or with media deprived of glucose (0 mM), as indicated for 24 h. IL-8 was measured by ELISA. Results are expressed as Mean ± SEM (*N* = 4). **G** A549 cells were plated for 24 h at normal conditions. Cells were then incubated at 0.1 or 21% of O_2_ in the hypoxic chamber for 24 hours. Supernatant were collected and secreted IL-8 measured. Results are expressed as Mean of fold change of pg of IL-8 / mg of proteins from cells in culture ± SEM (*N* = 3). **H** A549 cells were pretreated with 80 nM Actinomycin D (ActD) for 1 h; then cells were cultured in glucose-rich media in presence or absence of ActD (80 nM) supplemented with 10% dFBS for the indicated time points (0.5–6 h). Secreted IL-8 was measured by ELISA. Results are expressed as Mean ± SEM (*N* = 3). **I** A549 cells were pretreated with either TNFα (10 ng/ml + Glc 25 mM), glucose (25 mM) or without glucose (0 mM) for 3 h. Then media was replaced with fresh treatment media also containing ActD (80 nM) for times shown (0.25–4 h). *CXCL8* mRNA decay was measured for each condition. Results are expressed as Mean fold change ± SEM (*N* = 3, except for TNFα that is equal to 2). Statistical analysis was performed for **A**–**D** using a paired *t*-test comparing to the shortest time point, for **G** compared to 21% O_2_ G+, for **H** each time point ActD versus control sample. *p*-value: *p < 0.05, **p < 0.01, ***p < 0.001.

Human lung cancer cells with different mutations secrete high amounts of IL-8 under normal growing conditions, and murine lung cancer cells secrete the IL-8 ortholog KC (Fig.1A–E, Fig S1A). To investigate what drives IL-8 production, we first analyzed its secretion in the lung adenocarcinoma cell line A549 cultured under different conditions. TNFα, a well-known stimulus for IL-8 production, promotes its release (Fig. 1F). In addition, cells grown without glucose secreted more IL-8, as we have previously described in a variety of cell lines [13] (Fig. 1F). The presence of growth factors regulated basal and starvation-induced levels of IL-8, which was higher with increasing concentrations of serum in culture (Fig. 1F). Hypoxia is another characteristic of the tumor microenvironment. Hypoxia, however, did not promote IL-8 secretion (Fig. 1G). *CXCL8* synthesis was stimulated at the mRNA level by medium replacement, both under glucose-deprived and glucose-rich conditions, possibly reflecting the addition of fresh nutrients and growth factors from serum under experimental conditions (Fig. S1B, C). Actinomycin D, an inhibitor of RNA polymerases, prevented IL-8 secretion and mRNA increase, both under glucose-rich and glucose-free conditions (Fig. 1H, Fig. S1B–D). To address whether the regulation of IL-8 mRNA upon medium change, glucose deprivation or TNFα treatment was due to changes in its half-life, we analyzed its decay 3 h after treatments and observed that the mRNA had a half-life of 31 min, which only changed slightly under glucose deprivation or TNFα treatment (Fig. 1I, Fig. S1E). Therefore, although IL-8 can be regulated at the protein level under nutrient starvation [25], IL-8 is dynamically regulated in culture by de novo mRNA synthesis, and the increase of IL-8 under glucose deprivation is mainly due to an increase in its transcription.

### NF-kappaB and MAP kinase activity promote IL-8 secretion

**Fig. 2:**
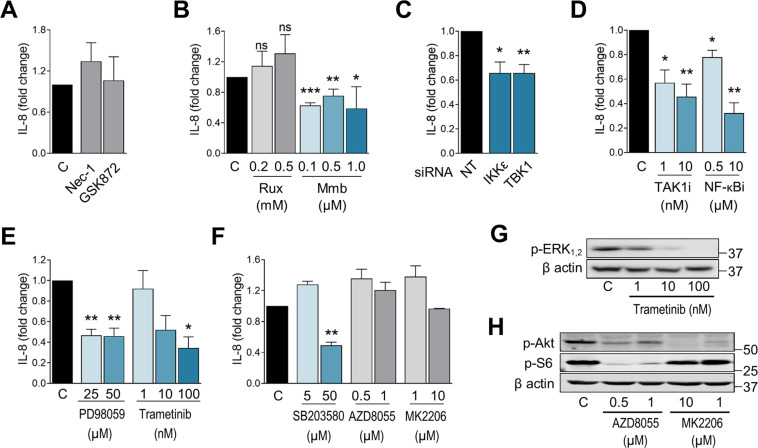
NF-kappaB and MAP kinase activity promote IL-8 secretion. **A** A549 cells were plated and incubated with 25 mM glucose media supplemented with 10% dFBS for 24 h. Medium was replaced for fresh medium containing Nec-1 at 20 µM or GSK872 at 1 µM for 16 h. Supernatants were collected and IL-8 concentration was measured by ELISA. Results are expressed as Mean of fold change ± SEM of at least two repeats. **B** Same as **A** but using two JAK1/2 inhibitors: Ruxolinitib (Rux) and Momelotinib (Mmb) at indicated concentrations. Results expressed as Mean of fold change ± SEM (N at least of 3). **C** A549 cells were transfected for 24 h with nontargeting siRNA (called’NT’) or siRNA for IKK-ε or for TBK1. Cells were left in culture for additional 24 h. IL-8 secretion was then analyzed through ELISA, expressed as Mean of fold change ± SEM (*N* = 3). **D** A549 cells were treated with Takinib (Tak inhibitor, TAK1-i) at 1 nM and 10 nM, with BAY11-7082 (NF-κB inhibitor, NF-κB-i) at 0.5 µM and 10 µM and for 16 h. IL-8 secretion was measured by ELISA, results are expressed as Mean of fold change ± SEM (*N* = 4). **E** A549 cells were treated with PD98059 (MAPKK inhibitor) at 25 μM and 50 μM; with Trametinib (MEK inhibitor) at 1, 10, and 100 nM for 16 h. IL-8 secretion was measured by ELISA, results are expressed as Mean of fold change ± SEM (N at least 3). **F** A549 cells were treated with SB203580 (p38 and Akt/ERK inhibitor) at 5 µM and 50 µM, AZD8055 (mTORC1 and mTORC2 inhibitor) at 0.5 µM and 1 µM, or with MK2206 (pan-inhibitor of Akt) at 1 µM and 10 µM for 16 h. IL-8 secretion was measured by ELISA, results are expressed as Mean of fold change ± SEM (N at least 3). **G** Representative western blot of p-ERK1/2 upon treatment as described in **E**. **H** Representative western blot of p-Akt and p-S6 upon treatment as described in **F**. Statistical analysis performed using Ratio paired *t*-test compared to the control sample. *p*-value: *p < 0.05, **p < 0.01, ***p < 0.001.

To identify signaling routes that mediate IL-8 gene expression and secretion, we employed inhibitors for kinases that regulate cytokine secretion in multiple cancer models. Some of these inhibitors are being tested in clinical trials for cancer, or are used in the clinic for inflammatory disease [26–31]. We observed that inhibition of JAK1/2 using Ruxolitinib (INCB18424), RIPK1 using Necrostatin-1s, and RIPK3 using GSK872 did not reduce IL-8 secretion at basal levels (Fig. 2A, B) or under starvation (Fig. S2A).

In contrast, BAY11-7082 (NF-κB inhibitor), PD98059 (MAPKK MEK inhibitor), Trametinib (MEK inhibitor), Takinib (Tak-1 inhibitor), SB203580 (p38 and Akt/ERK inhibitor), and Momelotinib reduced IL-8 secretion both at basal levels and under glucose starvation (Fig. 2B, D and Fig. S2A, C). Momelotinib is a JAK1/2 and TBK1/IKK-ε inhibitor that reduces CCL5 and IL-6 production in NSCLC [31]. Results shown in Fig. 2B using Ruxolitinib, a different JAK1/2 inhibitor, suggest that the effects of Momelotinib in modulating IL-8 production were not related to the JAKs but to TBK1/IKK-ε, which are known off-targets of this inhibitor. This was confirmed by knocking down these kinases under normal and starvation conditions (Fig. 2C and Fig. S2B).

BAY11-7082 is an inhibitor of NF-κB activation, and this transcription factor regulates IL-8 synthesis in multiple contexts. We have previously shown that treatment with this drug, or the abrogation of the NF-κB subunit p65 strongly reduced IL-8 release from A549 cells under glucose starvation [13]. We show here that this inhibitor also had an effect under basal conditions (Fig. 2D and Fig. S2C). Additionally, Takinib, which inhibits the kinase TAK1 that acts upstream of NF-κB and MEK1/2 [32], prevented IL-8 release (Fig. 2D).

MEK inhibitors have been shown to reduce IL-8 induction in Ras-driven tumors [16]. MEK and the downstream ERK kinases regulate the release of multiple other cytokines from NSCLC [33, 34]. For this reason, we employed two MEK inhibitors, PD98059 and Trametinib, and observed that they inhibited ERK phosphorylation and prevented IL-8 release in A549 cells (Fig. 2E, G and Fig. S2C). SB203580 has been shown to reduce IL-8 production in response to radiation in lung cancer cells, an effect that was attributed to the p38-MAPK pathway [35]. We employed this drug which has later been described to have effects on p38, PKB(Akt), and ERK pathways. As shown in Fig. 2F and Fig. S2C, only higher dosage of SB203580 blocks IL-8 secretion, suggesting an involvement of the PKB-Akt or ERK rather than the p38 pathway. The Akt and mTOR pathways were probed by direct inhibition of the three main Akt isoforms with MK2206, and by blocking mTORC1 and mTORC2 activity with AZD8055. These inhibitors had no effect on IL-8 production (Fig. 2F, H). In summary, the NF-κB, TAK1, TBK1/IKK-ε, and MEK/ERK MAP kinase pathways participate in IL-8 secretion by A549 cells, but not Akt, mTOR or JAK kinases.

### NF-κB regulates IL-8 production under basal and stimulated conditions

**Fig. 3:**
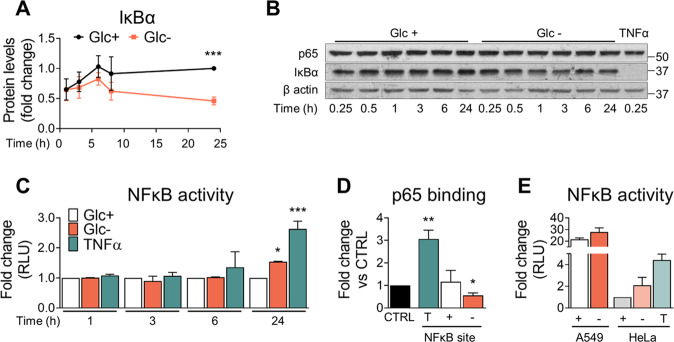
NF-κB regulates IL-8 production under basal and stimulated conditions. **A** A549 cells were treated with media with or without glucose (25–0 mM). Cells were collected at indicated time points to analyze p65 and IĸBɑ by western blot. Results are shown compared to 24 h Glc + and are expressed as Mean of fold change ± SEM (*N* > 4). **B** Representative western blot of **A** is shown. **C** A549 cells were transfected with a NF-κB reporter plasmid based on luciferase and treated with media containing either TNFα (10 ng/ml + Glc 25 mM, called ‘T’), glucose (25 mM, called ‘+’) or without glucose (0 mM, called ‘−’) for indicated time points, in 1% dFBS. Cells were lysed and luminescence measured for each sample. Results are shown compared to Glc + samples of each time point. Mean of fold change ± SEM (*N* = 3). **D** A549 cells were treated for 6 h with media containing either TNFα (10 ng/ml + Glc 25 mM, indicated as ‘T’), glucose (25 mM, ‘+’) or deprived of glucose (0 mM, ‘−’). Cells were collected and nuclear proteins extracted. p65 binding to NF-ĸB site in *CXCL8* promoter was measured by qDPI-ELISA. Results are shown compared to CTRL. Mean of fold change ± SEM (*N* = 4). **E** A549 and HeLa cells were treated with media containing either TNFα (10 ng/ml + Glc 25 mM, called ‘T’), glucose (25 mM, called ‘+’) or without glucose (0 mM, called ‘−’) for 24 h. Cells were lysed and luminescence measured for each sample. Results are shown compared to HeLa cells treated with glucose media, mean of fold change ± SEM (*N* = 2). Statistical analysis performed using Ratio paired *t*-test. *p*-value: *p < 0.05, **p < 0.01, ***p < 0.001.

We had previously observed that the higher expression of IL-8 under glucose-deprived conditions was in part due to upregulation of ATF4, a transcription factor of the Unfolded Protein Response (UPR), and of the canonical NF-κB pathway [13], (Fig. 2D). Besides being highly expressed under starvation, ATF4 also regulated IL-8 production under basal conditions (see Fig. S2D for a diagram of *CXCL8* promoter). Additionally, NF-κB regulates the production of IL-8 in multiple cancer cell lines, including A549 [13]. The UPR, as well as treatment with TNFα, also stimulates NF-κB signaling, at least in part due to the translational arrest that reduces the levels of the short-lived NF-κB inhibitor IκBα [36–38]. Therefore, we examined the possibility that NF-κB was further activated upon glucose depletion. The NF-κB protein inhibitor IκBα, which keeps the transcription factor inactive, was completely degraded upon TNFα treatment and modestly but significantly reduced at 24 h of glucose deprivation (Fig. 3A, B) [37, 38]. At shorter time points upon glucose removal, no differences were observed (Fig. 3A, B). An increase in NF-κB activation at 24 h was corroborated by NF-κB-driven luciferase activity measurement (Fig. 3C). However, at short time points (3 or 6 h), when IL-8 is transcriptionally induced by glucose deprivation (ref. 8 and Fig. S1C, D), we could not detect changes in NF-κB activity by luciferase assays, nor in p65 binding to the NF-κB binding site in IL-8 promoter (Fig. 3D). A549 cells, like most cancer cells, show high constitutive activity of NF-κB (Fig. 3E), thereby masking a potential differential activation between these two conditions. In summary, in addition to ATF4, the NF-κB, MAPK, and TBK1/IKK-ε pathways are involved in basal, TNF-induced and starvation-induced IL-8 secretion (Fig. 2C, D and S2B, C).

### TRAIL death receptors mediate IL-8 secretion

**Fig. 4:**
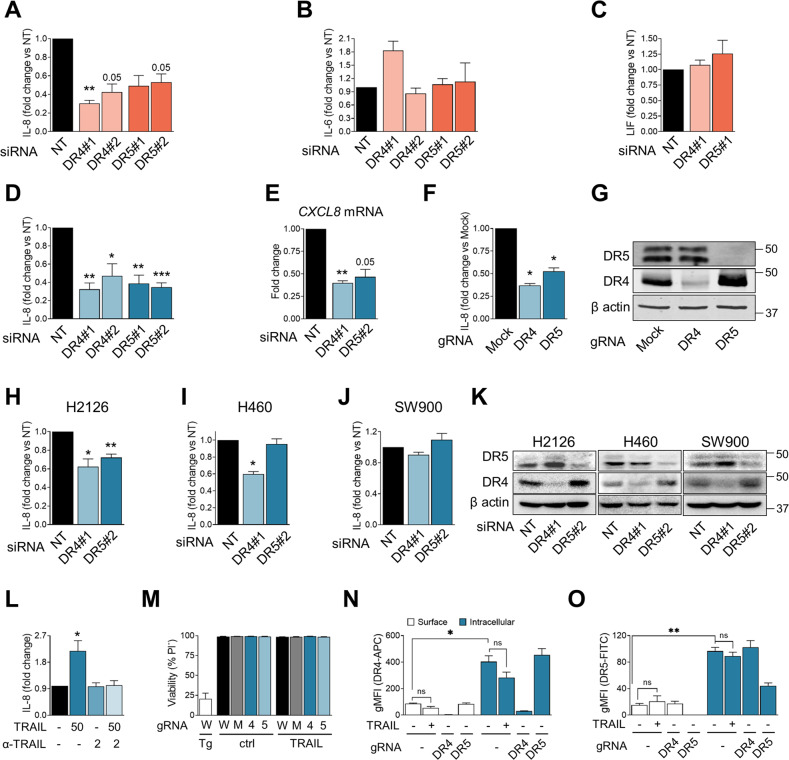
TRAIL death receptors mediate IL-8 secretion. **A**–**C** A549 cells were transfected for 24 h with nontargeting siRNA (labeled as “NT”) or siRNA for DR4 (“DR4#1” for first sequence and “DR4#2” for second sequence) or siRNA for DR5 (“DR5#1” for first sequence and “DR5#2” for second sequence). Media was changed for fresh glucose-free media and supernatants were collected 16 h later. IL-8 secretion (**A**), IL-6 (**B**), and LIF (**C**) was measured via ELISA. Results are expressed as Mean of fold change ± SEM (*N* = 3). **D** As in **A**, but media was changed for fresh rich-glucose media. Mean of fold change ± SEM (*N* = 3). **E** A549 cells were transfected for 24 h with nontargeting siRNA (“NT”) or siRNA for DR4 (“DR4#1”) or siRNA for DR5 (“DR5#2”). Media was changed and left it for 16 more hours. Cells were collected 16 h after medium replacement, and IL-8 expression was measured via qPCR, shown as Mean of fold change ± SEM (*N* = 3). **F**, **G** DR4, DR5 were CRISPRed out in A549 cells. After plating the cells, media was changed and cells were incubated for 24 h. Supernatants were collected and IL-8 secretion was measured (**F**). Results are shown normalized to basal mock. Statistic comparison was made vs their own mock sample. Results are represented as Mean of fold change ± SEM (*N* = 4). A representative WB is shown in **G**. **H**–**K** H2126, H460, and SW900 cells were transfected for 24 h with nontargeting siRNA (labeled as “NT”) or siRNA for DR4 (labeled “DR4#1”) or siRNA for DR5 (labeled “DR5#2”). Media was changed for fresh one and left it for 24 more hours replaced, and supernatants were collected and 24 h later. IL-8 secretion was measured via ELISA. Results shown as Mean of fold change ± SEM (*N* = 3) for H2126 cells (**H**), H460 (**I**), and SW900 (**J**). Representative western blots of DR4 and DR5 upon silencing of DR4 and DR5 are shown in **K**. **L** A549 cells were treated with TRAIL (50 ng/ml), antibody anti-TRAIL (2 ng/ml) or their combination for 24 h. Supernatants were collected and IL-8 secretion was measured. Results are expressed as Mean of fold change ± SEM (N at least of 3). **M** A549 WT (called “W”), Mock (called “M”), DR4 KO (called “4”) and DR5 KO (called “5”) cells were plated for 24 h and media was replaced with DMEM, DMEM containing 20 µM Thapsigargin as positive control of cell death (Tg) or DMEM containing 50 ng/ml TRAIL. Cells were incubated for 24 h and then collected for PI staining. Cell viability was measured by FACS and indicated as percentage of cells which are PI^−^. Results are expressed as Mean ± SEM (*N* = 3). **N** A549 WT, DR4 KO and DR5 KO cells were plated for 24 h. Media was changed to fresh DMEM or fresh DMEM with TRAIL (50 ng/ml) as indicated for an additional 24 h. Cells were collected and stained for surface or total DR4. Cells were measured by FACS and intracellular staining was calculated by subtracting the gMFI of the surface staining to the gMFI of the total staining. Results are shown as gMFI ± SEM (*N* = 3). **O** Same as **N** but for DR5. Statistical analysis performed using Ratio paired *t*-test compared to NT, or to Mock basal level for **F**. *p*-value: *p < 0.05, **p < 0.01, ***p < 0.001.

The TBK1, TAK1, MAP kinases, and NF-κB pathways respond to activation of receptors of the TNF receptor superfamily. Among these proteins, the TRAIL receptors DR4 and DR5 are highly expressed in Human lung cancer [39]. Additionally, our lab and others have shown that these receptors are induced by glucose deprivation [40] and therefore could potentially be involved in the inflammatory response mediated by nutrient starvation. For these reasons, we investigated the role of TRAIL death receptors in IL-8 production in lung cancer cells. By transiently knocking down DR4 or DR5 in A549 cells (Fig. S3A–C), we observed that these receptors participated in glucose starvation-induced IL-8 production and secretion (Fig. 4A). In contrast, other cytokines secreted by starved A549 cells, such as IL-6 and LIF, did not show any reduction by removing the TRAIL receptors (Fig. 4B, C). Unexpectedly, the reduced production and secretion of IL-8 was also observed in cells grown in regular, glucose-containing DMEM (Fig. 4D, E). To confirm the results, we generated clones deficient in each of the TRAIL receptors via CRISPR. Analysis of basal IL-8 secretion in these clones confirmed that both DR4 and DR5 promoted basal IL-8 release in culture (Fig. 4F, G). Moreover, silencing DR4 or DR5 also downregulated IL-8 release in other lung adenocarcinoma cells like H2126 (Fig. 4H, K). DR4 regulated IL-8 release in H460 cells (Fig. 4I, K). SW900, a squamous cell carcinoma cell line, did not display regulation of IL-8 by TRAIL death receptors (Fig. 4J, K).

TRAIL receptors can be activated without TRAIL, particularly in the context of endoplasmic reticulum stress [40, 41]. TRAIL expression was undetectable in the four NSCLC cell lines, both by ELISA and western blot (Fig. S4A, B). A TRAIL neutralizing antibody abrogated IL-8 induction by recombinant TRAIL but did not reduce the constitutive IL-8 release by A549 cells (Fig. 4L), showing dissociation between the TRAIL ligand and IL-8 secretion. To further explore the possible TRAIL involvement, TRAIL levels were analyzed by qPCR, and the amplification cycles required for A549 cells were >30 in the presence or absence of glucose (Fig. S4C). For this reason, siRNA experiments were performed in H2126 cells in which TRAIL was detectable by qPCR and also showed DR4 and DR5 dependent IL-8 production (Fig. 4H). These experiments suggested that TRAIL was not involved in IL-8 production (Fig. S4D, E). On the other hand, as already described [18], TRAIL did not reduce the viability of A549 cells or TRAIL receptor knocked-out A549 by 24 h (Fig. 4M). To further understand the mechanism of signaling of the death receptors, their location was studied, identifying DR4 and DR5 to be mainly located intracellularly in the presence or absence of TRAIL (Fig. 4N, O).

### TRAIL receptor associated pathways regulate basal IL-8 secretion

**Fig. 5:**
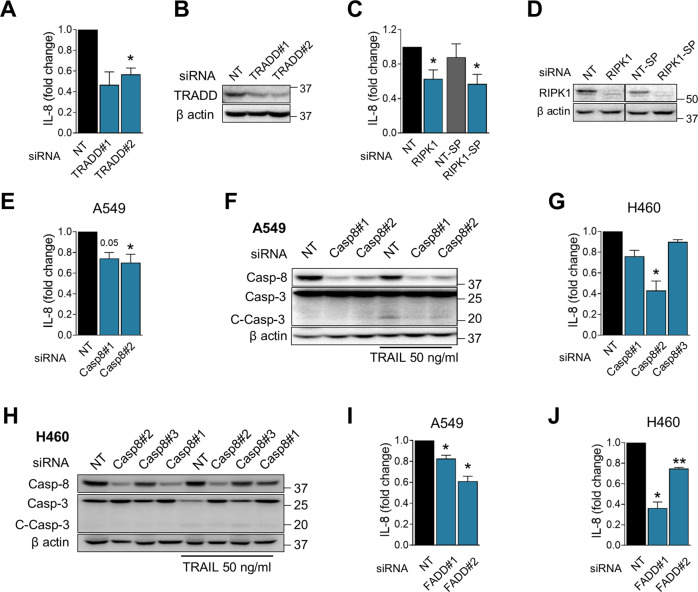
TRAIL receptor associated molecules regulate basal IL-8 secretion. **A** A549 cells were transfected with nontargeting siRNA (“NT”) or siRNA for TRADD (“TRADD#1” for first sequence and “TRADD#2” for second sequence) for 48 h. Media was changed and cells left in culture for an additional 24 h. Supernatants were collected and IL-8 measured. From the same samples, proteins were collected and silencing was validated through WB. Results are expressed as fold change of secreted IL-8 in siTRADD vs NT. Mean of fold change ± SEM (*N* = 3). **B** Western blot of TRADD silencing in A549 cells. A representative western blot is shown. **C** Same as **A**, but using siRNA for RIPK1. Smart pool siRNA was also used (“RIPK1-SP” with its control “NT-SP”). Results are expressed as fold change of secreted IL-8 in siRIPK1 vs NT-ctrl and RIPK1-SP vs NT-SP. Results are expressed as Mean of fold change ± SEM (*N* = 3). **D** Western blot of RIPK1 silencing in A549 cells. A representative western blot is shown. **E** Same as **A**, but using siRNA for Caspase-8 (“Casp8#1” first sequence and Casp8#2” second sequence). Results are expressed as fold change of secreted IL-8 in siCaspase8 vs NT. Mean of fold change ± SEM (*N* = 4). **F** Representative western blot of Caspase-3 cleavage and Caspase-8 in A549 cells with siRNA against Caspase-8. Cells were treated with or without media containing TRAIL (50 ng/ml) for 24 h after transfection for 48 h. A representative western blot is shown. **G** Same as **E** but in H460 cells (“Casp8#1” first sequence and”Casp8#2” second sequence”Casp8#3” third sequence). Results are expressed as fold change of secreted IL-8 in siCaspase-8 vs NT. Mean of fold change ± SEM (N at least 3). **H** Representative western blot of Caspase-3 cleavage and Caspase-8 in H460 cells with siRNA against Caspase-8. Cells were treated with or without media containing TRAIL (50 ng/ml) for 24 h after transfection for 48 h. A representative western blot is shown. **I** Same as **A**, but silencing FADD. (“FADD#1” for first sequence and “FADD#2” for second sequence). Results are expressed as fold change of secreted IL-8 in siFADD vs NT. Mean of fold change ± SEM (*N* = 3). **J** Same as I but in H460 cells. Results are expressed as fold change of secreted IL-8 in siFADD vs NT. Mean of fold change ± SEM (*N* = 3). Statistical analysis performed using a one-way ANOVA, multiple comparison test compared to their NT. In **C** a Ratio paired *t*-test analysis compared to NT or NT-SP were performed. *p*-value: *p < 0.05, **p < 0.01, ***p < 0.001.

TRAIL receptors, in response to exogenous TRAIL, regulate cytokine expression through TRADD in A549 cells [18]. Under non-stimulated conditions, we observed that TRADD silencing reduced constitutive secretion of IL-8 (Fig. 5A, B). TRADD seemed to participate directly downstream of TRAIL receptors but not TNFR1, since these cells did not secrete TNFα even after treatment with TRAIL (Fig. S5A) and silencing TNFR1 only very slightly reduced basal IL-8 production (Fig. S5B, C).

TRAIL activates NF-κB via the FADDosome, an intracellular complex composed of RIPK1, FADD, and Caspase-8, which acts in a catalytically independent manner [17]. Silencing RIPK1 significantly decreased IL-8 secretion from A549 cells (Fig. 5C, D) even though the RIPK1 inhibitor Necrostatin-1s did not (Fig. 2A). The roles of FADD and caspase-8 were more difficult to ascertain. Downregulation of Caspase-8 using siRNA produced modest results on IL-8 under basal conditions in A549 (Fig. 5E, F), which were more evident in H460 cells (Fig. 5G, H) and under TRAIL treatment (Fig. S5D, E). siRNA against Caspase-8 was, however, sufficient to reduce Caspase-3 activation upon treatment with TRAIL (Fig. 5F, H). The reduction in FADD levels using siRNA partially prevented basal and/or TRAIL-induced IL-8 release in A549 and H460 (Fig. 5I, J, Fig. S5F–I), but some toxicity was observed with the siRNA and particularly when employing a dominant negative form of FADD (not shown), which may be due to necroptosis or to mitotic functions of FADD in Ras-driven NSCLC [42]. H2126 cells secreted the same amounts of IL-8 with or without TRAIL and with or without FADD (Fig. S5J, K).

**Fig. 6:**
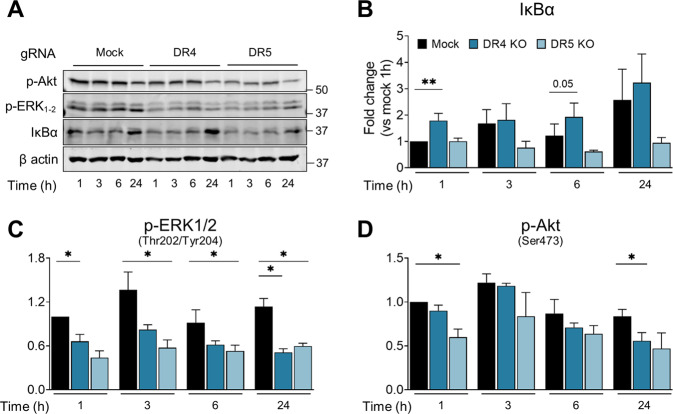
TRAIL receptor associated signaling pathways regulate basal IL-8 secretion. **A** Representative western blot of IκBα, p-ERK1/2, and p-Akt upon DR4 and DR5 CRISPRed out in A549 cells. Cells were plated for 24 h and media was replaced. Cells were collected at the indicated time points and proteins extracted for western blot analysis to measure total IκBα (densitometry shown in **B**), p-ERK1/2 (shown in **C**), and p-Akt (shown in **D**). Results indicate the values normalized to the internal control β-actin and compared to basal mock condition at 1 h, expressed as Mean of fold change ± SEM (N at least 3). Statistical analysis performed using a Ratio paired *t*-test vs. Mock. *p*-value: *p < 0.05, **p < 0.01, ***p < 0.001.

Stimulation with TRAIL activates several pro-survival pathways like NF-κB, PI3K/Akt and several MAP kinases in different cell types [43–48]. Both NF-κB and MAP kinases were involved in IL-8 secretion in A549 cells (Fig. 2D, E and Fig. S5C). We aimed to identify which of these pathways were maintained by tonic activation of TRAIL receptors, with or without TRAIL. Stimulation with TRAIL only mildly activated NF-κB and only at long time points; a much weaker effect than observed by its family member TNFα which completely depleted IκBα (Fig. 3B, Fig. S6A, B). Interestingly, analysis of IκBα levels as a surrogate of the NF-κB pathway activity indicated that DR4 regulates the constitutive activation of NF-κB, as DR4-deficient cells expressed more IκBα under basal conditions (Fig. 6A, B). In contrast, the absence of DR5 increased NF-κB activation. This suggests that DR4 and DR5, contribute antithetically to basal stimulation of NF-κB in A549 cells.

Another major regulator of IL-8 release in lung cancer is the MEK/ERK pathway, which is constitutively active in some cell lines with mutations in Ras or EGFR pathways, or activated by growth factors. Intriguingly, both TRAIL receptors regulated MAP kinase activity in Ras-mutated A549 cells (Fig. 6A, C). The absence of either receptor decreased the basal phosphorylation levels of ERK1/2. This effect was observed 1 h after medium change, and it was maintained over time in culture (Fig. 6A, C). Moreover, TRAIL did not induce ERK phosphorylation in these cells, supporting that this effect is TRAIL-independent (Fig. S6A, C). The same effects of DR4 and DR5 on ERK phosphorylation were observed when the receptors were downregulated transiently using siRNA (Fig. S6E). In addition to these, the absence of DR4 and DR5 decreased basal Akt phosphorylation on Ser473 (Fig. 6A, D), an mTORC2 target residue essential to fully activate Akt [49]. This suggests activation of the PI3K/Akt-mTOR pathway downstream of the TRAIL receptors, as previously observed downstream of DR5 in A549 cells [21]. This kinase, however, is not involved in IL-8 secretion according to results in Fig. 2 using inhibitors. Altogether, these data point towards DR4 and DR5 participating in IL-8 secretion through regulation of NF-κB (by DR4) and ERK-MAPK (by both TRAIL receptors).

### TRAIL receptors correlate with IL-8, angiogenesis, and neutrophil expression markers in human tumors

**Fig. 7:**
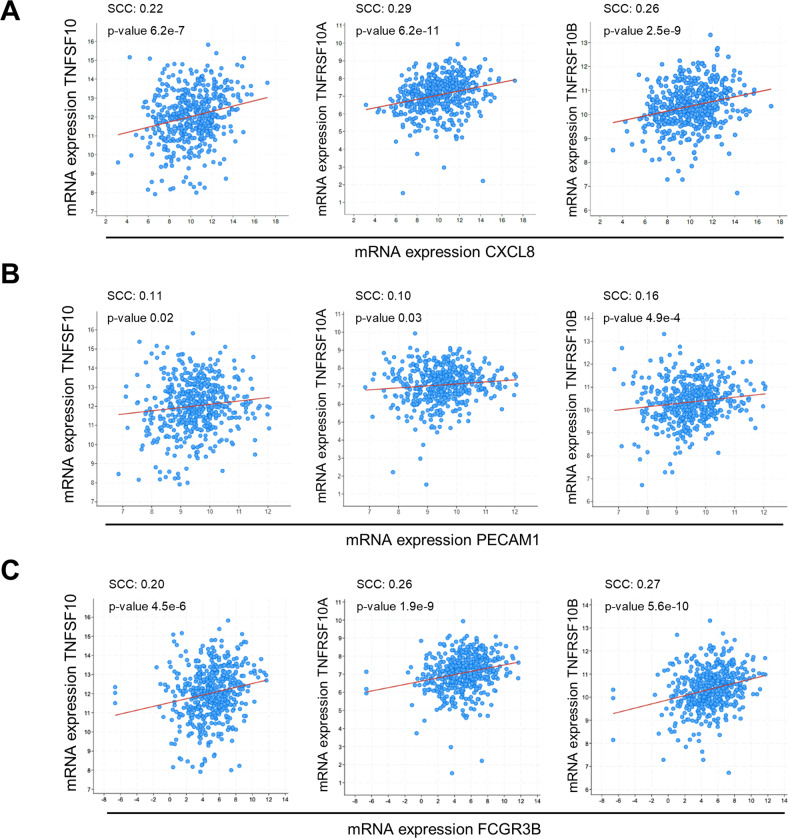
TRAIL receptors correlate with, IL-8, angiogenesis and neutrophil expression markers in Human lung squamous cell carcinoma. **A** Correlation data for TRAIL (TNSFSF10), DR4 (TNFRSF10A), and DR5 (TNFRSF10B) versus IL-8 (CXCL8) log2 mRNA expression are shown. mRNA expression z-scores relative to all samples of the Lung Squamous Cell Carcinoma (LUSC) TCGA, Firehose Legacy dataset was used. The statistical significance was determined using the Spearman Correlation Coefficient (SCC) in cBioportal online software. **B** Same as **A** but compared to the angiogenesis marker CD31 (PECAM-1). **C** Same as **A** but compared to the neutrophil marker CD16b (FCGR3B).

IL-8 is thought to contribute to lung cancer progression by favoring neo-angiogenesis, possibly indirectly via neutrophil chemotaxis. We interrogated TCGA database and assessed whether expression of TRAIL, DR4, and DR5 are related to IL-8 expression, angiogenesis, and neutrophil abundance. TRAIL, DR4 and DR5 mRNAs independently correlated with IL-8 expression in lung squamous cell carcinoma (Fig. 7A) and to a lower extent, in lung adenocarcinoma (Fig. S7A). The endothelial marker PECAM-1 (CD31), a surrogate for blood vessel density, correlated with TRAIL, DR4, and DR5 levels in LUSC patients, although the association was low (Fig. 7B). In LUAD, PECAM-1 only significantly correlated with TRAIL (Fig. S7B). The neutrophil marker CD16B was significantly associated with expression of DR4, DR5 and TRAIL in LUSC (Fig. 7C and with TRAIL and DR4 in LUAD (Fig. S7C). Altogether, and in line with the poor prognosis associated with TRAIL and TRAIL receptor expression [21, 50], these data suggest that TRAIL death receptors contribute to Human lung cancer tumorigenesis.

## Discussion

**Fig. 8:**
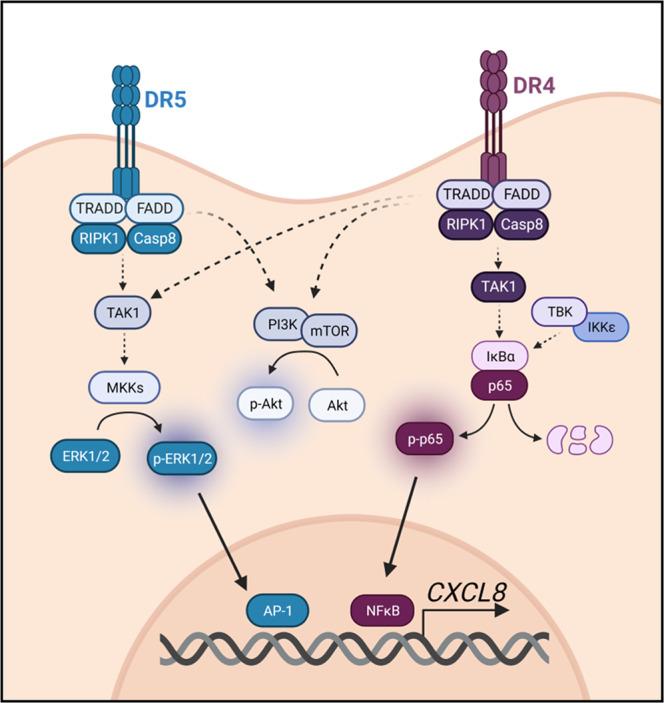
Diagram of IL-8 regulation via TRAIL death receptors 4 and 5. DR4 and DR5 activate IL-8 production through the MEK MAP kinases and AP-1. Death receptor downstream signaling includes TRADD, FADD, RIPK1 and Caspase-8. DR4 additionally regulates NF-kB. Both receptors regulate the PI3K-AKT pathway but this has no apparent role in IL-8 production.

In this study we show that several lung cancer cells with diverse mutational Ras status and WT EGFR secrete the chemokine IL-8 in non-stimulated conditions. The data show that IL-8 mRNA is short-lived, and therefore, this basal secretion is partially controlled by constant de novo transcription of *CXCL8* messenger RNA. Kinase inhibition experiments identified several main regulators: NF-κB, MEK-MAPK, TAK1, and TBK1/IKK-ε. This brought us to postulate that DR4 and DR5 could be upstream factors of this process. The characterization of signaling pathways downstream of TRAIL receptors has been thoroughly investigated after treatment with a biotinylated ligand, subsequent immunoprecipitation of TRAIL with bound receptors, and determination of recruitment of molecules over time. However, the fact that DR-dependent IL-8 secretion occurs without the addition of TRAIL, and therefore without acute stimulation, complicates the dissection of the components of the DR4 and DR5 signalosome in the absence of a tagged ligand. TRAIL receptors engage multiple signaling pathways by recruitment of proteins into intracellular platforms like the FADDosome, the DISC, and the ripoptosome. Using transient silencing, we found involvement of RIPK1 and, unexpectedly, TRADD, in IL-8 secretion. TRADD has been found in other settings to act downstream of TRAIL for pro-inflammatory functions or cell death signaling [18, 51]. Having excluded the relevance of TNF receptor 1, and given that TNFα was not secreted constitutively or after TRAIL treatment in A549 cells, we suggest a possible role of TRADD directly downstream of TRAIL receptors, or alternatively, bound to other cooperating TNF receptor superfamily members. Caspase-8 and FADD downregulation had modest effects on IL-8 release and they were not fully consistent between different cell lines, which may suggest that the downregulation achieved using siRNA may not be sufficient to determine their role in the pathway, although our results point towards a participation of both proteins in IL-8 release (see diagram in Fig. 8).

We examined the signaling mechanisms downstream of DR4 and DR5 connected to IL-8 release and identified two signaling branches. NF-κB, most probably coupled with TBK1/IKK-ε, TAK1, and the FADDosome, was triggered only by DR4 but nor DR5, while the MAP kinases were controlled by both receptors 4 and 5 (Fig. 8). The link between NF-κB and IL-8 secretion upon several diverse stimuli has already been described [17, 35, 52], and MAPK activation has been shown to affect IL-8 induction in Ras-driven tumors [16]. However, there are no other studies that link the interaction of death receptors and these signaling pathways to IL-8 secretion in unstimulated conditions. TRAIL receptor activation in absence of TRAIL has also been described elsewhere [17, 40, 53–55]. Accordingly, we demonstrate that TRAIL neutralization did not affect the secretion of IL-8, and moreover, TRAIL stimulation did not affect the localization of the receptors, nor did induce the ERK-MAPK, PI3K-Akt, or NF-κB pathways. This leads us to speculate that DR4 and DR5 could be activating these pathways from an intracellular location rather than from the cell surface, as described by Lam et al. [56]. Another possibility would be that these receptors are coupled to other receptors of the same family, and they act together by being activated by a different ligand secreted by the cells or present in the serum, although our data exclude TNFR1 as this possible partner. As a third possibility, bovine serum may contain components that activate TRAIL receptors, including possibly trace amounts of bovine TRAIL. Serum did potentiate IL-8 secretion, which could alternatively be due to modulation of protein synthesis or to further activation of MAP kinases or NF-κB by growth factors like EGFR.

The mouse genome does not contain *CXCL8*, which has limited the studies of regulation of IL-8 secretion in the TME. Murine orthologs of *CXCL8* with functions that partially overlap with pro-tumorigenic IL-8 functions are CXCL1/KC and CXCL2/MIP2, which promote mouse tumor growth and angiogenesis [57]. Consistent with the described role of IL-8, we show that *CXCL8* expression correlates significantly with endothelial cells (CD31) and neutrophils (CD16b). *CXCL8* correlates as well with the expression of DR4 (TNFRSF10A) and DR5 (TNFRSF10B) in NSCLC. As already shown [18], TRAIL expression positively correlates with other tumor-associated markers in LUAD patients, suggesting a role for the ligand in tumorigenesis. Along with others [17, 51, 58], we show that TRAIL does induce IL-8, but also that the receptors are capable of stimulating intracellular signals in a ligand-independent manner. Therefore, TRAIL is not necessary for the tumor cells employed in our study to secrete IL-8. TRAIL expression (TNFSF10) significantly correlated with *CXCL8* expression in the LUSC but not the LUAD dataset, suggesting that the ligand may be participating in IL-8 release in some patients. More studies, particularly multivariate analysis, as well as analysis of TRAIL levels in patient sera, would be required to explore the role of TRAIL in patients.

TRAIL death receptors have long been shown to specifically kill tumor cells without harming non-cancerous ones. However, multiple reports pointed a dual role for death receptors: stimulating pro- or anti-tumorigenic signaling [17, 21]. Of note, the murine TRAIL receptor was described to be responsible of CCL2 secretion which in turn promotes a supportive TME via alternatively activated myeloid cells (MDSCs) [18]. Additionally, TRAIL receptors mediate inflammation in response to chemotherapy [17]. This evidence indicates that there are crucial aspects missing to fully characterize the tumor supportive role of TRAIL receptors. In this context, activation of death receptors by death ligands such as TNFα, Fas ligand or TRAIL may be counterproductive if cells undergo cytokine release rather than death [17, 18]. Given the inflammatory and pro-angiogenic role of IL-8, our results suggest that caution is needed when stimulating therapeutically TRAIL receptors in cancer patients.

## Supplementary information

Supplementary Figure 1

Supplementary Figure 2

Supplementary Figure 3

Supplementary Figure 4

Supplementary Figure 5

Supplementary Figure 6

Supplementary Figure 7

Supplementary Tables 1 and 2

Cell line authentication

Original Data File

## Data Availability

All data generated or analyzed during this study are included in this published article and its supplementary information files.
