# Supplementary figures and images for: TRAIL receptors promote constitutive and inducible IL-8 secretion in non-small cell lung carcinoma
Source: Cell Death Dis. 2022 Dec 15;13(12):1046. doi: 10.1038/s41419-022-05495-0 (PMC9755151; doi:10.1038/s41419-022-05495-0)

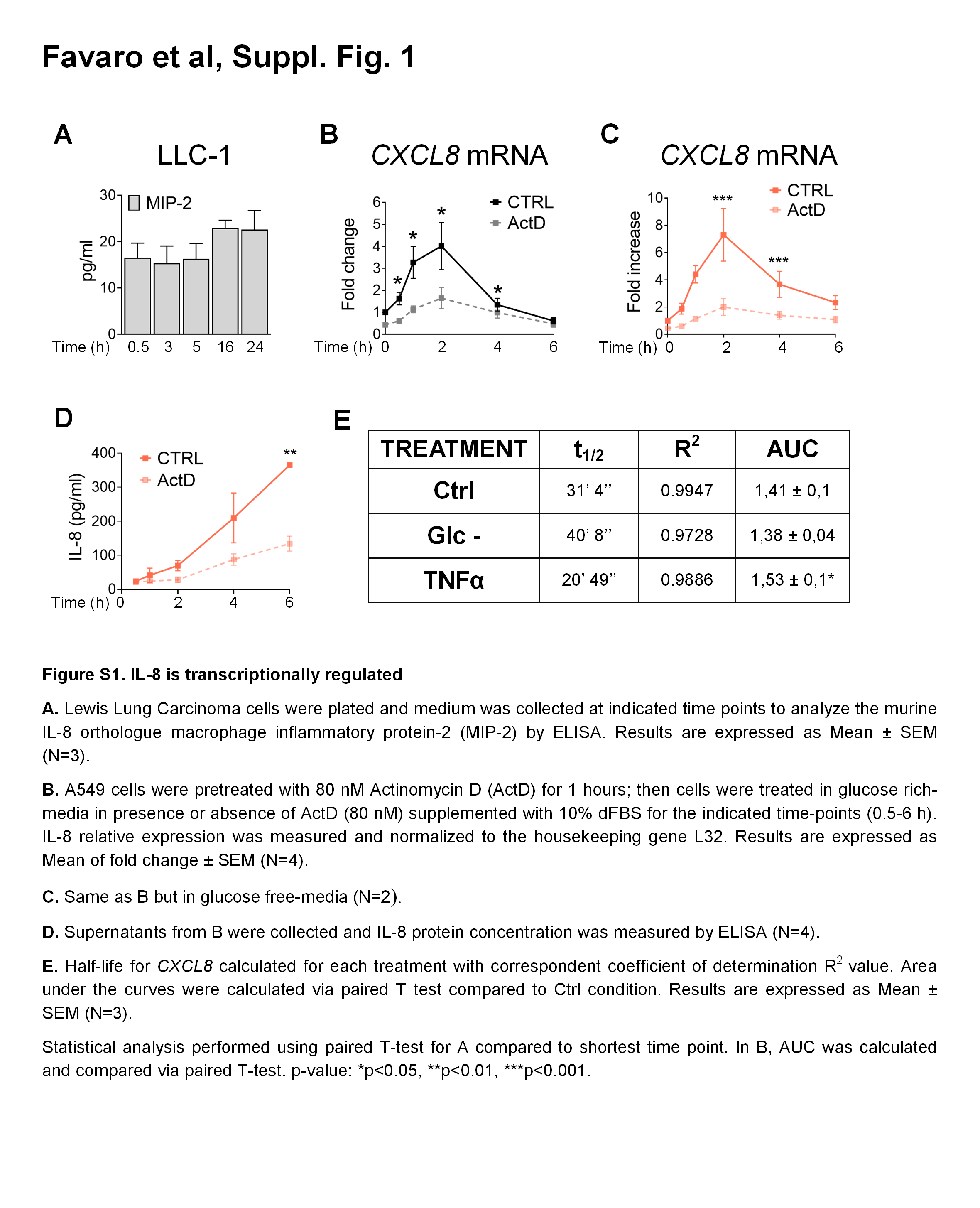

Supplement: Supplementary file 1 — Supplementary Figure 1 [file 41419_2022_5495_MOESM1_ESM.tif]

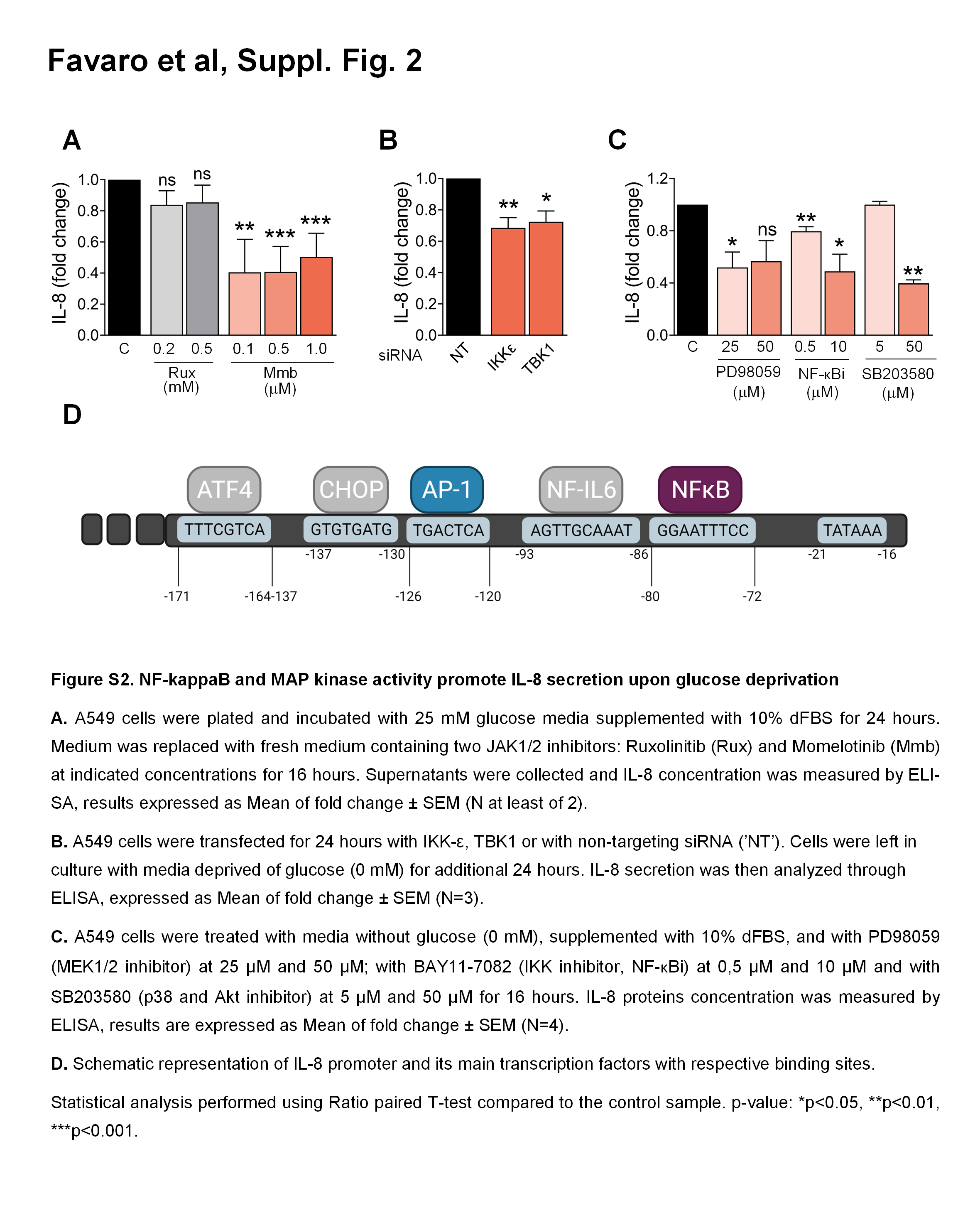

Supplement: Supplementary file 2 — Supplementary Figure 2 [file 41419_2022_5495_MOESM2_ESM.tif]

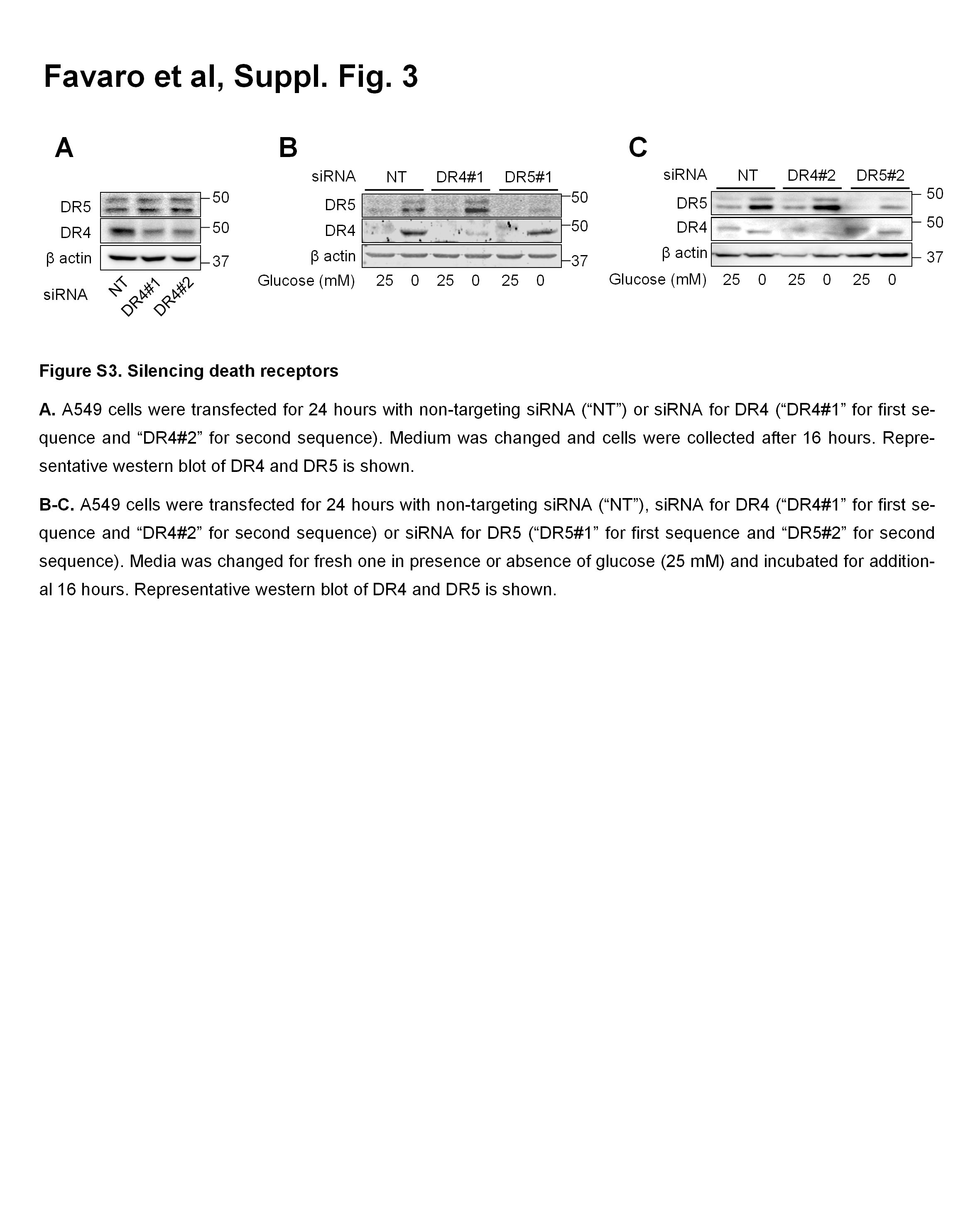

Supplement: Supplementary file 3 — Supplementary Figure 3 [file 41419_2022_5495_MOESM3_ESM.tif]

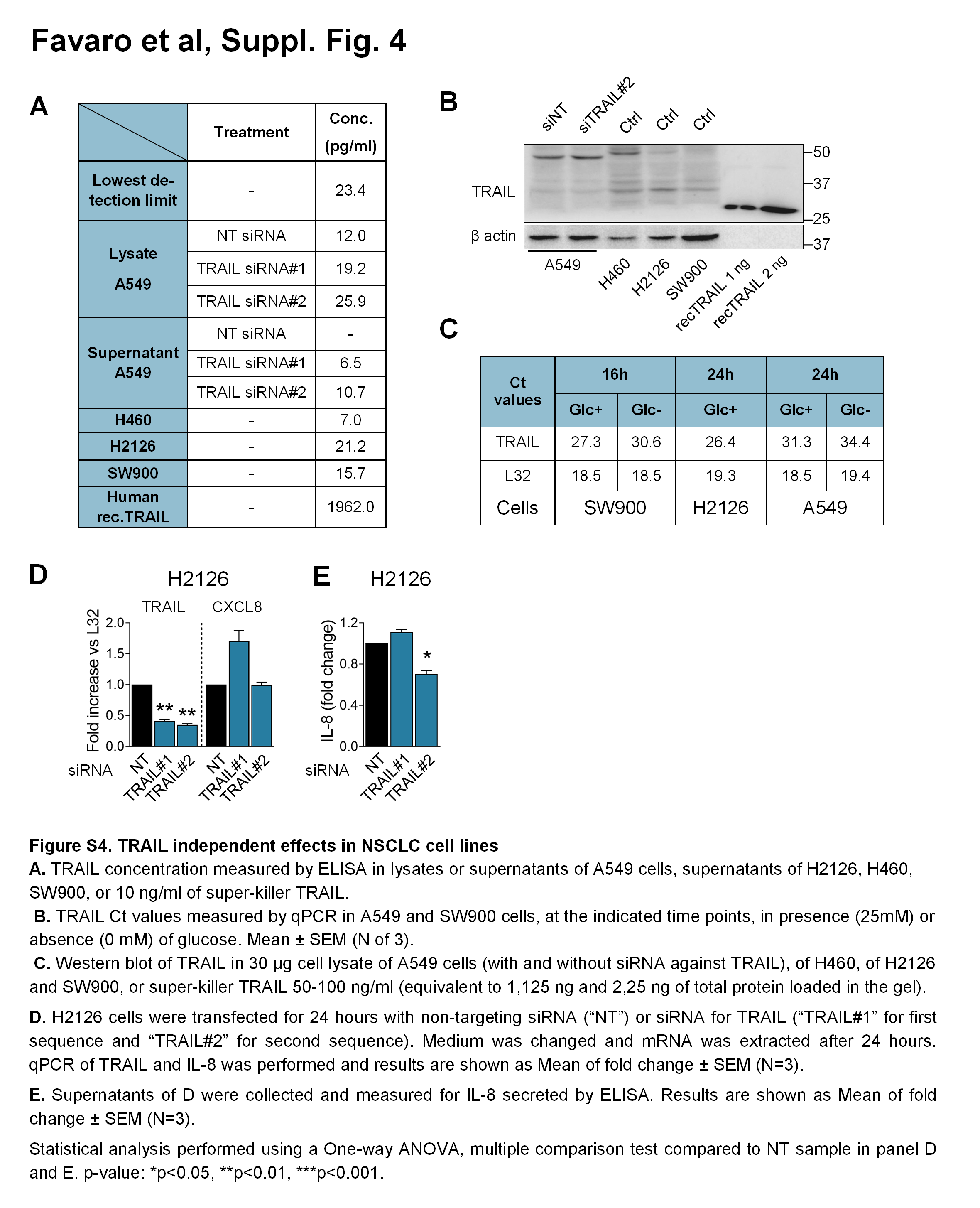

Supplement: Supplementary file 4 — Supplementary Figure 4 [file 41419_2022_5495_MOESM4_ESM.tif]

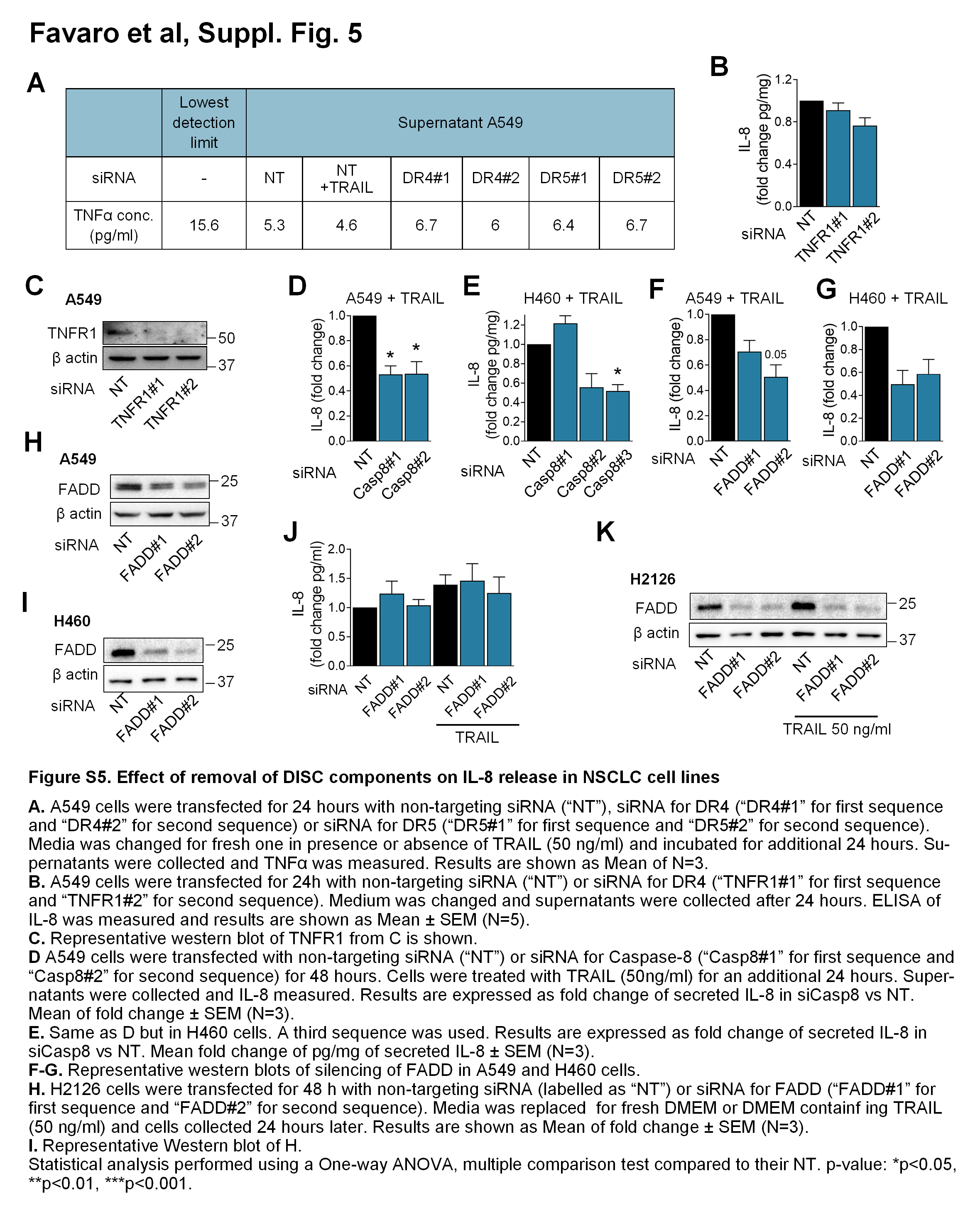

Supplement: Supplementary file 5 — Supplementary Figure 5 [file 41419_2022_5495_MOESM5_ESM.tif]

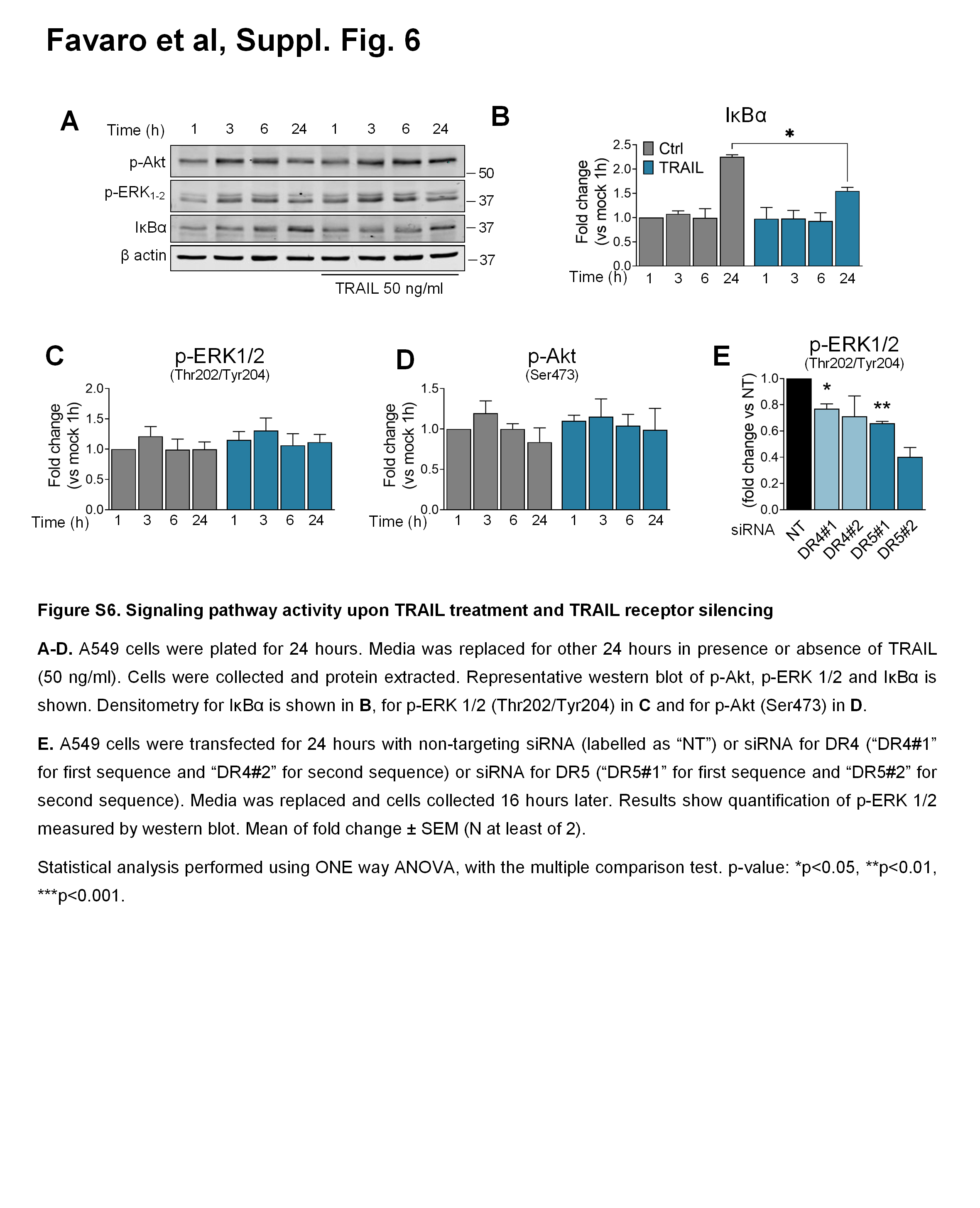

Supplement: Supplementary file 6 — Supplementary Figure 6 [file 41419_2022_5495_MOESM6_ESM.tif]

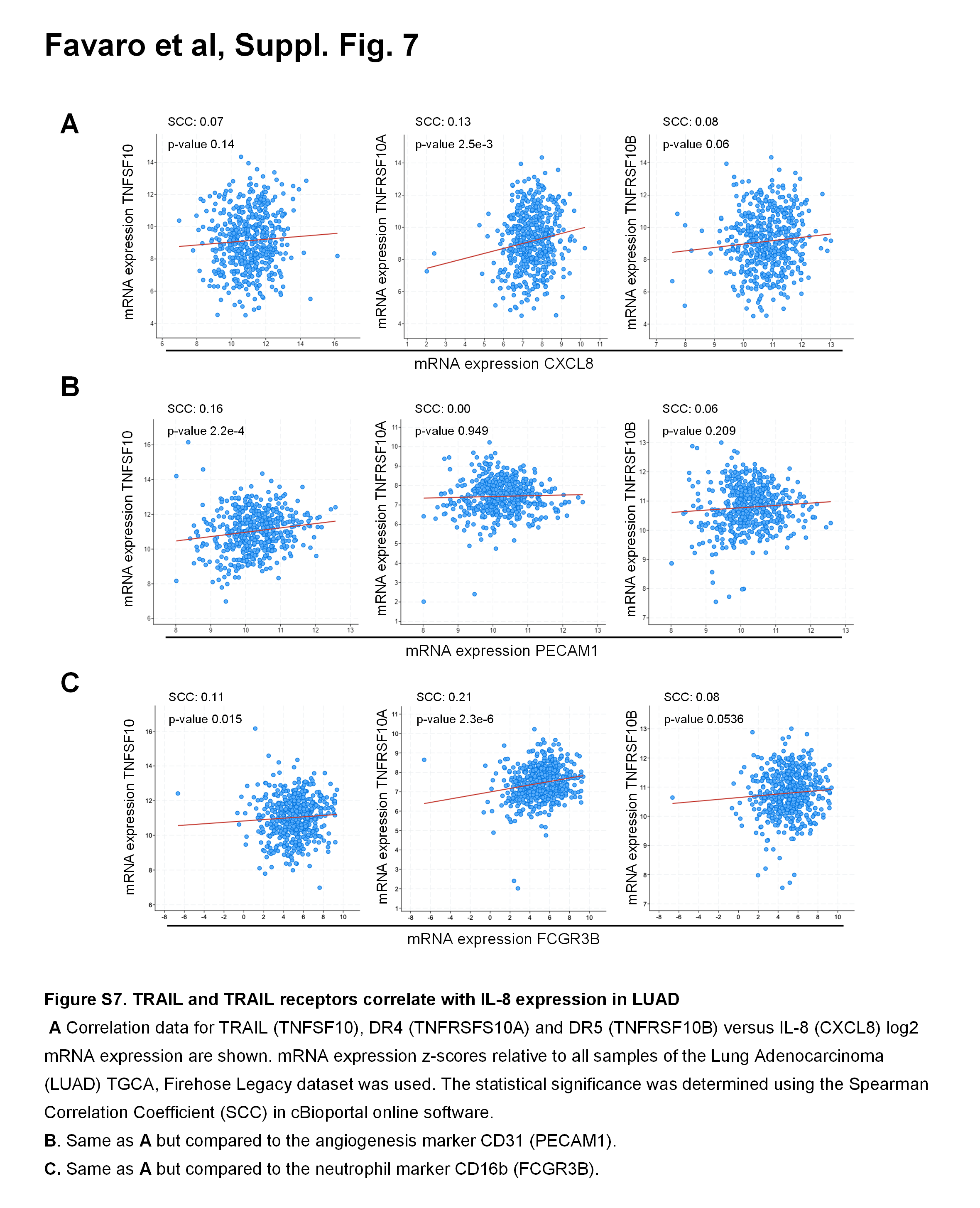

Supplement: Supplementary file 7 — Supplementary Figure 7 [file 41419_2022_5495_MOESM7_ESM.tif]
