# Supplementary material for: TRAIL receptors promote constitutive and inducible IL-8 secretion in non-small cell lung carcinoma
Source: Cell Death Dis. 2022 Dec 15;13(12):1046. doi: 10.1038/s41419-022-05495-0 (PMC9755151; doi:10.1038/s41419-022-05495-0)
Supplement: Supplementary file 8 — Supplementary Tables 1 and 2 [file 41419_2022_5495_MOESM8_ESM.pdf]

Favaro et al, Suppl. Table 1. Antibody list

REAGENTS	SOURCE	IDENTIFIER
Antibodies		
b-actin (C4)	Merck Millipore	#MAB1501R
Caspase 3	Cell Signaling	#9662
Caspase 8	Abcam	#ab138485
FADD (H10)	Santa Cruz	#sc-271520
IκBα	Cell Signaling	#4814
p65 (D14E12)	Cell Signaling	#8242
p-Akt (Ser473)	Celll Signaling	#9271
p-ERK 1/2	Cell Signaling	#9101
RIPK1	BD Pharmingen	#610458
TNFR1	Santa Cruz	#sc-8436
TRADD	Santa Cruz	#sc-7868
TRAIL	BD Pharmingen	#556468
TRAIL-R1	Diaclone	#852.980.000
TRAIL-R1(D9S1R) *	Cell Signaling	#42533
TRAIL-R1 APC	Invitrogen	#MA5-28607
TRAIL-R2 (D4E9)	Cell Signaling	#8074S
TRAIL-R2 FITC	Invitrogen	#MA1-19759

*Antibody used only for validation of CRISPR-cas9 in A549 cells.

Favaro et al, Suppl. Table 2. Oligonucleotide list and sequences

Primers for qPCR	Forward 5’>3’	Reverse 3’>5’
CXCL8	5’-TTCACCAAATTGTGGAGCTTCAGT-3’	5’-CCTCAGGGCAAACCTGAGTCATC-3’
L32	5’-AACGTCAAGGAGCTGGAAG-3’	5’-GGGTTGGTGACTCTGATGG-3’
TNFSF10 (or TRAIL)	5’-GTCTCTCTGTGTGGCTGTAAC -3’	5’-GGGCTGTTCATACTCTCTTCG-3’
siRNA and gRNA	Forward 5’>3’	SOURCE
Non-Targeting (NT)	5’- UAAGGCUAUGAGAGAUAC [dt][dt] -3’	Designed by and ordered in Sigma
NT-Smart Pool (SP) – OnTargetPlus siRNA pools	Cat#D-001810-01-05	Designed by and ordered in Dharmacon
Casp8#1	5’- GGAGCUGCUCUUCCGAAUU -3’	Designed by and ordered in Sigma
Casp8#2	5’- AACUACCAGAAAGGUAUACCU -3’	Sullivan et al. 2020 Dev Cell
Casp8#3	5’- GUUCCUGAGCCUGGACUAC -3’	Sullivan et al. 2020 Dev Cell
FADD#1	5’- GAUUGGAGAAGGCUGGCUC [dT][dT] -3’	Sullivan et al. 2020 Dev Cell
FADD#2	5’- GAACUCAAGCUGCGUUUAU [dT][dT] -3’	Designed by and ordered in Sigma
IKKε	5’- GGUCUUCAACACUACCAGC[dT][dT] -3’	Designed by and ordered in Sigma
RIPK1	5’- CCACUAGUCUGACGGAUAAtt -3’	Designed by and ordered in Sigma
RIPK1-SP – OnTargetPlus siRNA pools	Cat#L-004445-00-005	Designed and ordered in Dharmacon
TNFR1#1	5’- GGAACCUACUUGUACAAUGAC-3’	Changhui et al. 2009 BioMed Res Int
TNFR1#2	5’- GCUGUGGACUUUUGUACAU-3’	Guo et al. 2014 Protein Cell
TRADD#1	5’- GGAGGAUGCGCUGCGAAAUUU[dT][dT] -3’	Sullivan et al. 2020 Dev Cell
TRADD#2	5’- CUGGCUGAGCUGGAGGAUG[dA][dA] -3’	Sullivan et al. 2020 Dev Cell
TRAIL#1	5’- AACGAGCUGAAGCAGAUGCAG -3’	Designed by and ordered in Sigma
TRAIL#2	5’- UUGUUUGUCGUUCUUUGUGUU -3’	Designed by and ordered in Sigma
TRAIL-R1 (or DR4) #1	5’- CACCAAUGCUUCCAACAAU -3’	Designed by and ordered in Sigma
TRAIL-R1 (or DR4) #2	5’- AACGAGAUUCUGAGCAACGCA -3’	Designed by and ordered in Sigma
TRAIL-R2 (or DR5) #1	5’- GCUGUGGAGGAGACGGUGAUU -3’	Designed by and ordered in Sigma
TRAIL-R2 (or DR5) #2	5’- GACCCUUGUGCUCGUUGUCdTdT -3’	Designed by and ordered in Sigma
TBK1	5’- GAACGUAGAUUAGCUUAUAUU -3’	Designed by and ordered in Sigma
gRNA for TRAIL-R1 (DR4)	Seq#1 5’-GGGAGGATTGAACCACGAGG-3’<br>Seq#2 5’- GAGGCAAGCAAACAAATTGT-3’<br>Seq#3 5’- AGGTCAAGGATTGTACGCCC-3’	Toronto KnockOut Library
gRNA for TRAIL-R2 (DR5)	5’- CCTACCGCCATGGAACAACG-3’	DeskGen Series CRISPR Library
Oligos for DPI	Forward 5’>3’	Reverse 3’>5’
CXCL8 promoter-NFκB binding site	5’- TGTCAGAGGAAATTCCACGATTT-GCAACTGATGGCCCATCCCCTCAGGGCAAAC biotin tag-3’	5’- GTTTGCCCTGAGGGGATGGGCCATCAG-TTGCAAATCGTGGAATTTCCTCTGACA biotin tag-3’
CXCL8 promoter-halfCRE/CHOP binding site	5’- GAAAACTTTCGTCATACTCCGTATTTGA-TAAGGAACAAATAGGAAGTGTGATGACTCA biotin tag-3’	5’- TGAGTCATCACACTTCCTATTT-GTTCCTTATCAAATACGGAGTATGACGAAAGTTTTC biotin tag-3’
CXCL8 promoter-CRE binding site	5’- CCTGAGGGGATGGGCCATCAGTTGCAAATCGTG-GAATTTCCTCTGACATAATGA biotin tag-3’	5’- TCATTATGTCAGAGGAAATTCCACGATTT-GCAACTGATGGCCCATCCCCTCAGG biotin tag-3’
