# Supplementary material for: TRAIL receptors promote constitutive and inducible IL-8 secretion in non-small cell lung carcinoma
Source: Cell Death Dis. 2022 Dec 15;13(12):1046. doi: 10.1038/s41419-022-05495-0 (PMC9755151; doi:10.1038/s41419-022-05495-0)
Supplement: Supplementary file 9 — Cell line authentication [file 41419_2022_5495_MOESM9_ESM.pdf]

eurofins | Genomics

Eurofins Genomics Europe Applied Genomics GmbH, Anzinger Str. 7 a, D-85560 Ebersberg

Dr. Joaquim Moreno-Caceres
IDIBELL
Avinguda de la Gran Via de l'Hospitalet 199
08908 L'Hospitalet de Llobregat
Spain

Certificate
Cell Line Authentication Test
Order ID: 11107213341

Report date: 25.01.2021

Method:
DNA isolation was carried out from cell pellet (cell layer).
Genetic characteristics were determined by PCR-single-locus-technology.
16 independent PCR-systems D8S1179, D21S11, D7S820, CSF1PO, D3S1358, TH01, D13S317, D16S539, D2S1338, AMEL, D5S818, FGA, D19S433, vWA, TPOX and D18S51 were investigated.
(ASN-0002 core markers are colored grey, Thermo Fisher, AmpFlSTR® Identifiler® Plus PCR Amplification Kit)
In parallel, positive and negative controls were carried out yielding correct results.

Result:

**Client Sample Name**	H460 p3 021220	SW900	H2126 p7 021220	A549 p26 011220	H1299 p20	HeLa GL p20
**Sample Code**	CL00002828	CL00002829	CL00002830	CL00002831	CL00002832	CL00002833
D8S1179	12,12	11,16	12,12	13,14	10,13	12,13
D21S11	30,30	29,31	29,29	29,29	32.2,32.2	27,28
D7S820	9,12	11,12	8,9	8,11	10,10	8,12
CSF1PO	11,12	11,11	11,11	10,12	12,12	9,10
D3S1358	15,18	14,14	16,16	16,16	17,17	15,18
TH01	9.3,9.3	8,8	7,9.3	8,9.3	6,9.3	7,7
D13S317	13,13	8,8	12,14	11,11	12,12	12,14
D16S539	9,9	11,11	12,12	11,12	12,13	9,10
D2S1338	17,25	20,27	17,19	24,24	23,24	17,17
D19S433	14,14	14,14	14,14	13,13	14,14	13,14
vWA	17,17	16,16	17,17	14,14	16,18	16,18
TPOX	8,8	11,11	8,8	8,11	8,8	8,12
D18S51	13,15	19,19	14,14	14,17	16,16	16,16
AMEL	X,Y	X,X	X,X	X,X	X,X	X,X
D5S818	9,10	11,11	11,11	11,11	11,11	11,12
FGA	21,23	23,23	26,26	23,23	20,20	18,21

This report was created automatically and is therefore valid without a signature.

The laboratory is accredited acc. to **DIN EN ISO/IEC 17025:2005**. The accreditation applies only to the test methods specified in the accreditation certificate. All analyses have been carried out with greatest care and on the basis of state of the art scientific knowledge. The results refer solely to the analysed samples. The duplication and publication also in parts requires a written authorization by this laboratory. Our General Terms and Conditions apply exclusively and are available under eurofinsgenomics.com

ilac-MRA DAkkS Deutsche Akkreditierungsstelle D-PL-13372-01-00

Eurofins Genomics Europe Applied Genomics GmbH
Anzinger Straße 7 a
85560 Ebersberg
Germany

Tel.: +49 8092 8289-200
Fax: +49 8092 8289-201
Email: info-eu@eurofins.com
Web: eurofinsgenomics.com

Managing Directors: Dr. Michael Hadem, Dr. Peter Persigehl
Register Court Munich HRB 207710
VAT ID: DE815473648

HypoVereinsbank
IBAN: DE23 2073 0017 7000 0006 50
SWIFT: HYVEDEMME17
