# Supplementary material for: TRAIL receptors promote constitutive and inducible IL-8 secretion in non-small cell lung carcinoma
Source: Cell Death Dis. 2022 Dec 15;13(12):1046. doi: 10.1038/s41419-022-05495-0 (PMC9755151; doi:10.1038/s41419-022-05495-0)

Full length uncropped WB - Main Figures

Fig 2G

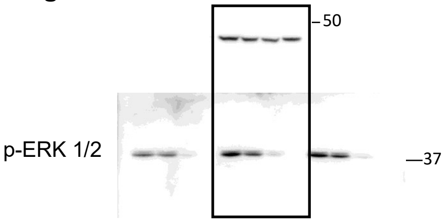

Fig 2H

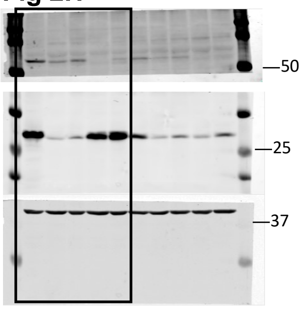

Fig 3B

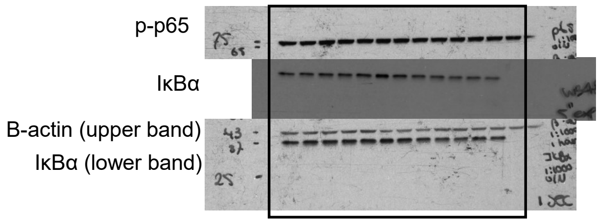

Fig 4G

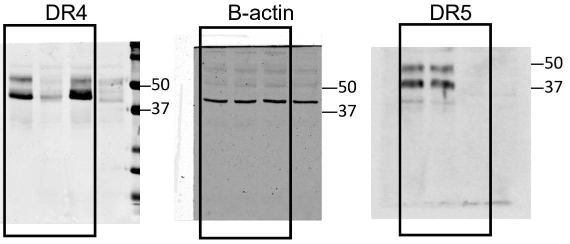

Fig 4K - H2126

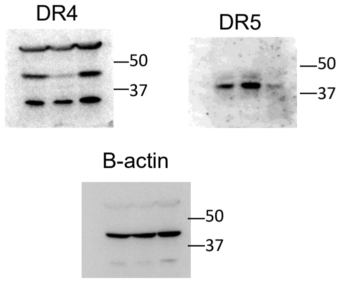

Fig 4K - SW900

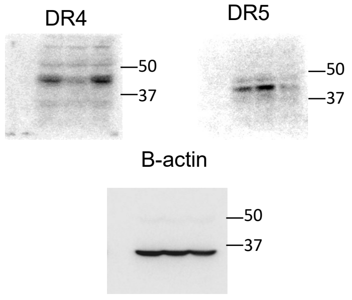

Fig 4K - H460

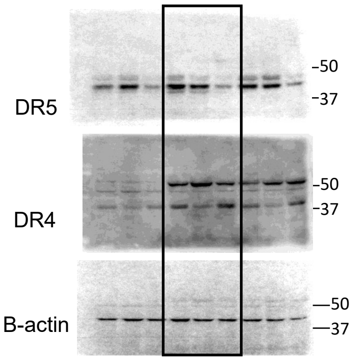

Full length uncropped WB - Main Figures

Fig 5B

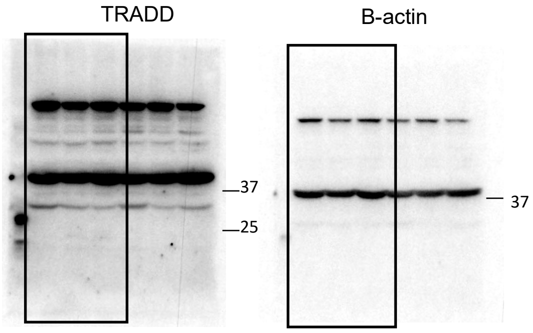

Fig 5D

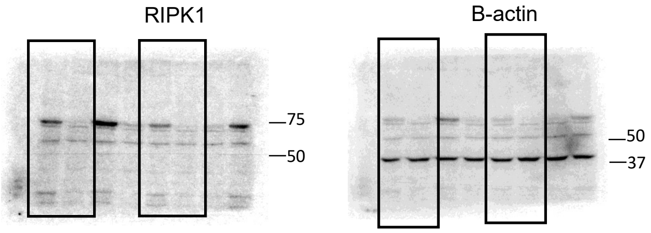

Fig 5F

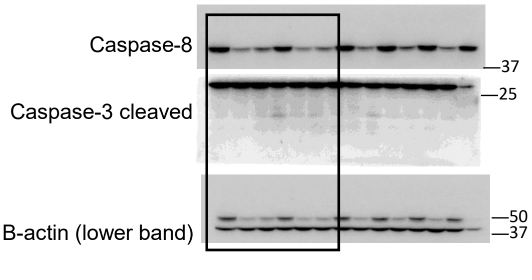

Fig 5H

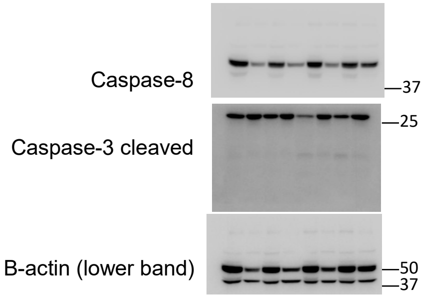

Fig 6A

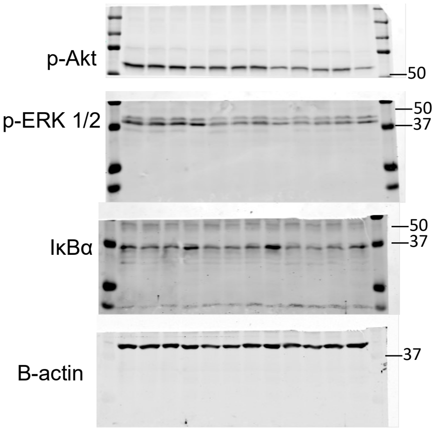

Full length uncropped WB - Supplementary Figures

Fig S3A

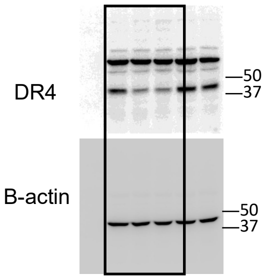

Fig S3B

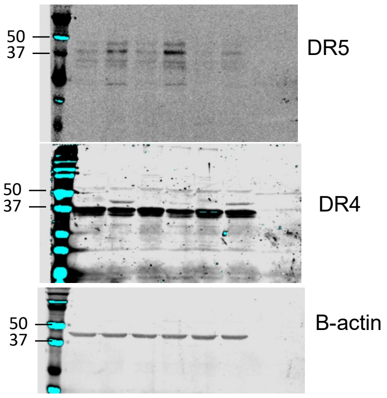

Fig S3C

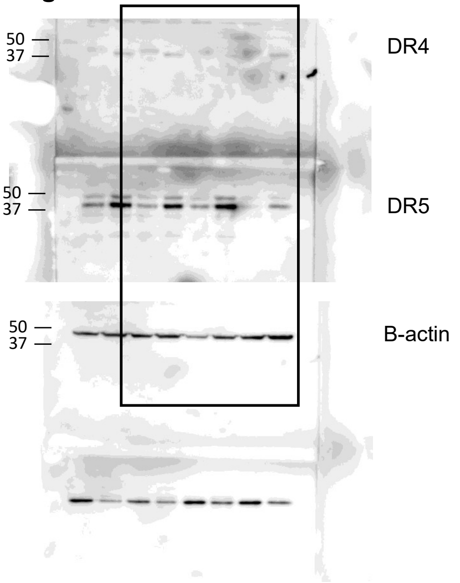

Fig S4B

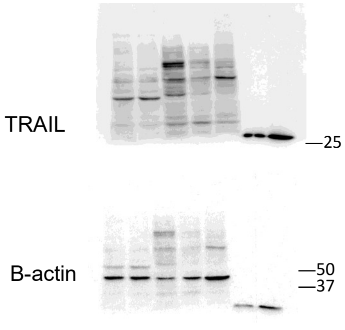

Fig S5C

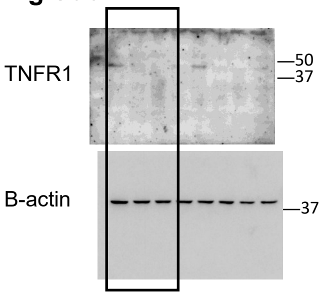

Fig S5H

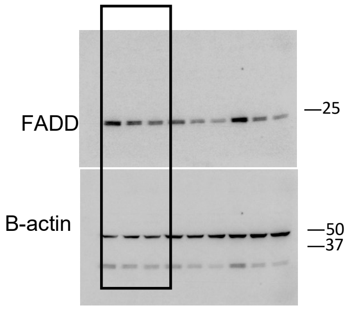

Fig S5I

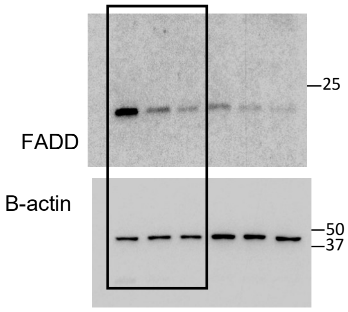

Fig S5K

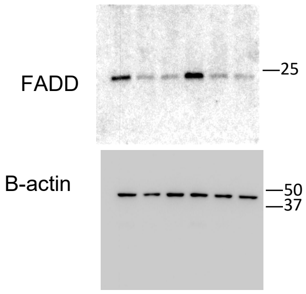

Fig S6A

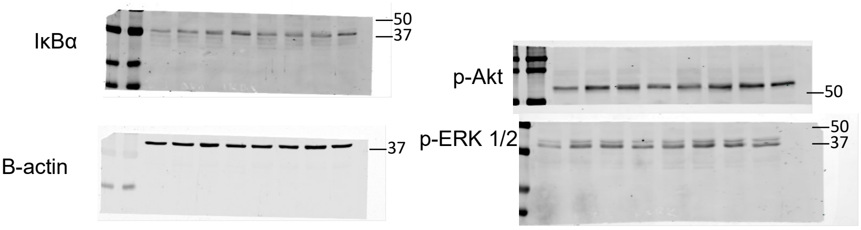

Supplement: Supplementary file 10 — Original Data File [file 41419_2022_5495_MOESM10_ESM.pdf]
